# Upregulated LAMA3 modulates proliferation, adhesion, migration and epithelial‑to‑mesenchymal transition of cholangiocarcinoma cells

**DOI:** 10.1038/s41598-023-48798-8

**Published:** 2023-12-18

**Authors:** Kittiya Islam, Brinda Balasubramanian, Simran Venkatraman, Parichut Thummarati, Janpen Tunganuntarat, Nut Phueakphud, Phongthon Kanjanasirirat, Tanawadee Khumpanied, Pornparn Kongpracha, Yingpinyapat Kittirat, Rutaiwan Tohtong, Tavan Janvilisri, Patompon Wongtrakoongate, Suparerk Borwornpinyo, Nisana Namwat, Tuangporn Suthiphongchai

**Affiliations:** 1 Department of Biochemistry, Faculty of Science, Mahidol University, Bangkok, 10400 Thailand; 2 Graduate Program in Molecular Medicine, Faculty of Science, Mahidol University, Bangkok, 10400 Thailand; 3 Department of Clinical Chemistry, Faculty of Allied Health Sciences, Chulalongkorn University, Bangkok, 10330 Thailand; 4 Excellent Center for Drug Discovery (ECDD), Faculty of Science, Mahidol University, Bangkok, 10400 Thailand; 5 Department of Biochemistry, Faculty of Medicine, Khon Kaen University, Khon Kaen, 40002 Thailand; 6 Cholangiocarcinoma Research Institute, Khon Kaen University, Khon Kaen, 40002 Thailand; 7 Present Address: Department of Medical Sciences, Regional Medical Sciences Center 2, Ministry of Public Health, Phitsanulok, 65000 Thailand; 8 Center for Neuroscience, Faculty of Science, Mahidol University, Bangkok, 10400 Thailand; 9 Department of Biotechnology, Faculty of Science, Mahidol University, Bangkok, 10400 Thailand; 10 Present Address: Department of Systems Biosciences and Computational Medicine, Faculty of Medicine, Khon Kaen University, Khon Kaen, 40002 Thailand

**Keywords:** Cancer, Cell biology, Computational biology and bioinformatics, Oncology

## Abstract

A poor outcome for cholangiocarcinoma (CCA) patients is still a clinical challenge. CCA is typically recognized by the desmoplastic nature, which accounts for its malignancy. Among various extracellular matrix proteins, laminin is the most potent inducer for CCA migration. Herein, we accessed the expression profiles of laminin gene family and explored the significance of the key laminin subunit on CCA aggressiveness. Of all 11 laminin genes, LAMA3, LAMA5, LAMB3 and LAMC2 were concordantly upregulated based on the analysis of multiple public transcriptomic datasets and also overexpressed in Thai CCA cell lines and patient tissues in which LAMA3A upregulated in the highest frequency (97%) of the cases. Differential expression genes (DEGs) analysis of low and high laminin signature groups revealed LAMA3 as the sole common DEG in all investigated datasets. Restratifying CCA samples according to LAMA3 expression indicated the association of LAMA3 in the focal adhesion pathway. Silencing LAMA3 revealed that it plays important roles in CCA cell proliferation, adhesion, migration and epithelial-to-mesenchymal transition. Taken together, this research signifies the roles of dysregulated ECM homeostasis in CCA malignancy and highlights, for the first time, the potential usage of LAMA3 as the diagnostic biomarker and the therapeutic target to tackle the CCA stromal.

## Introduction

Cholangiocarcinoma (CCA), the epithelial cancer of bile ducts, is relatively rare but notoriously lethal with the highest reported global incidence in northeastern Thailand. Combination of silent symptom with diagnostic delay, poor prognosis and chemoresistance contributes to an alarming raise in the global mortality of CCA yearly^1^. The aggressiveness of CCA is not only a consequence of the neoplastic cells themselves, but also a result of tumor microenvironment (TME) created by the extensive remodeling of extracellular matrix (ECM) constituents and the reciprocal communication between cancerous cells and the authoritative tumor stromal cells^2^. During CCA progression and metastasis, the surrounding ECM experiences significant biological and architectural alterations, including increased secretion of ECM proteins, such as collagens, fibronectins, laminins, periostin and tenascin-C^3^. These phenomena take part in the pathogenesis of CCA via several mechanisms involving in accelerating migration and invasion, evading immune destruction, and inducing angiogenesis^4^.

Understanding ECM roles and how disruption of ECM dynamics influences cancer development, and progression may shed the light on the improvement of early detection and promising treatment for patients with CCA. Laminins, a large family of heterotrimeric glycoproteins, assemble into cross-like structures from different combinations of five α (LAMA1–5), three β (LAMB1–3) and three γ (LAMC1–3) chains. At least 16 isoforms of laminins have been identified and accordingly named based on their chain composition; for instance, laminin-332 (formerly laminin-5) comprises α3, β3 and γ2 chains^5^. A wide variation in their biological roles is presumably mediated through their receptor repertoires, mainly integrins; for example, integrin α6β4 binding increase cell motility and proliferation through the activation of JNK cascade via Rac 1 or Ras/Raf/MAPK pathway, while integrin α6β1 binding causes tyrosine phosphorylation of p190RhoGAP, and thus stimulates protrusive and degradative activities necessary for invasion^6,7^. Copious amount of evidence has reported the aberrant expression of laminins and their association with biological characteristics and clinical outcomes of several types of cancers, such as colon, kidney, liver, lung and stomach^7^.

Epithelial-to-mesenchymal transition (EMT) is a reversible cellular program which transiently transforms epithelial cells into mesenchymal phenotypes and behaviors. During EMT, epithelial cells encounter specific signals from their microenvironment to trigger (i) the reorganization of cytoarchitecture into fibroblast-like morphology, (ii) the downregulated expression of intercellular junction proteins, such as claudin-1, E-cadherin and zonula occludens‑1 and (iii) the upregulation of mesenchymal markers, namely fibronectin, N-cadherin and vimentin. Concurrently, these phenomena loosen cell–cell adhesion, alter cell–matrix interaction and ameliorate migratory capacity, apoptosis resistance and ECM degradation, which are crucial processes for cancer metastasis^8^.

Alteration of the ECM in CCA plays several roles in malignancy behaviors. These roles include promoting cell proliferation, migration, metastasis, EMT, angiogenesis, fibrosis, and ECM stiffness. Additionally, it weakens basement membrane integrity, recruits inflammatory cells and myofibroblasts, and serves as reservoirs for cytokines and growth factors that affect cancer progress^9^. Several types of alterations, such as overexpression of periostin or tenascin C, correlate with metastasis and short survival of CCA patients^9^. Due to the complex relationship between TME and the enhanced malignant behaviors of CCA, the significance of ECM proteins in the pathogenesis of CCA still needs to be addressed. Among a variety of evaluated ECM proteins, laminin shows the highest ability in enhancing CCA cell migration and the overexpressed LAMB1 is correlated with lymphatic invasion^10^. Suppression of LAMB3 expression by microRNA‐329 inhibits EMT and lymph node metastasis in CCA^11^. Upregulation of LAMC2 expression in CCA is associated with CCA malignancy^12,13^. Up to our knowledge, however, relatively little is known about the expression of laminin gene family and their contribution to CCA progression as well as the potential usage as CCA biomarkers.

In the current study, we utilized multiple publicly available datasets containing transcriptomic data of CCA tissues to investigate the expression of laminin gene family. We also validated these results in tissues and cell lines of Thai CCA patients. Furthermore, we identified LAMA3 as the uniquely upregulated laminin in CCA and further explored its functions and a novel molecular mechanism of action in CCA progression. Our finding underlines the gravity of dysregulated cell-ECM interaction in CCA malignancy and suggests the optimistic prospect in using LAMA3 not only as the diagnosis biomarker for CCA but also as the therapeutic strategy for desmoplastic CCA therapy.

## Results

### Expression profiling of laminin gene family in CCA through publicly available databases

**Figure 1:**
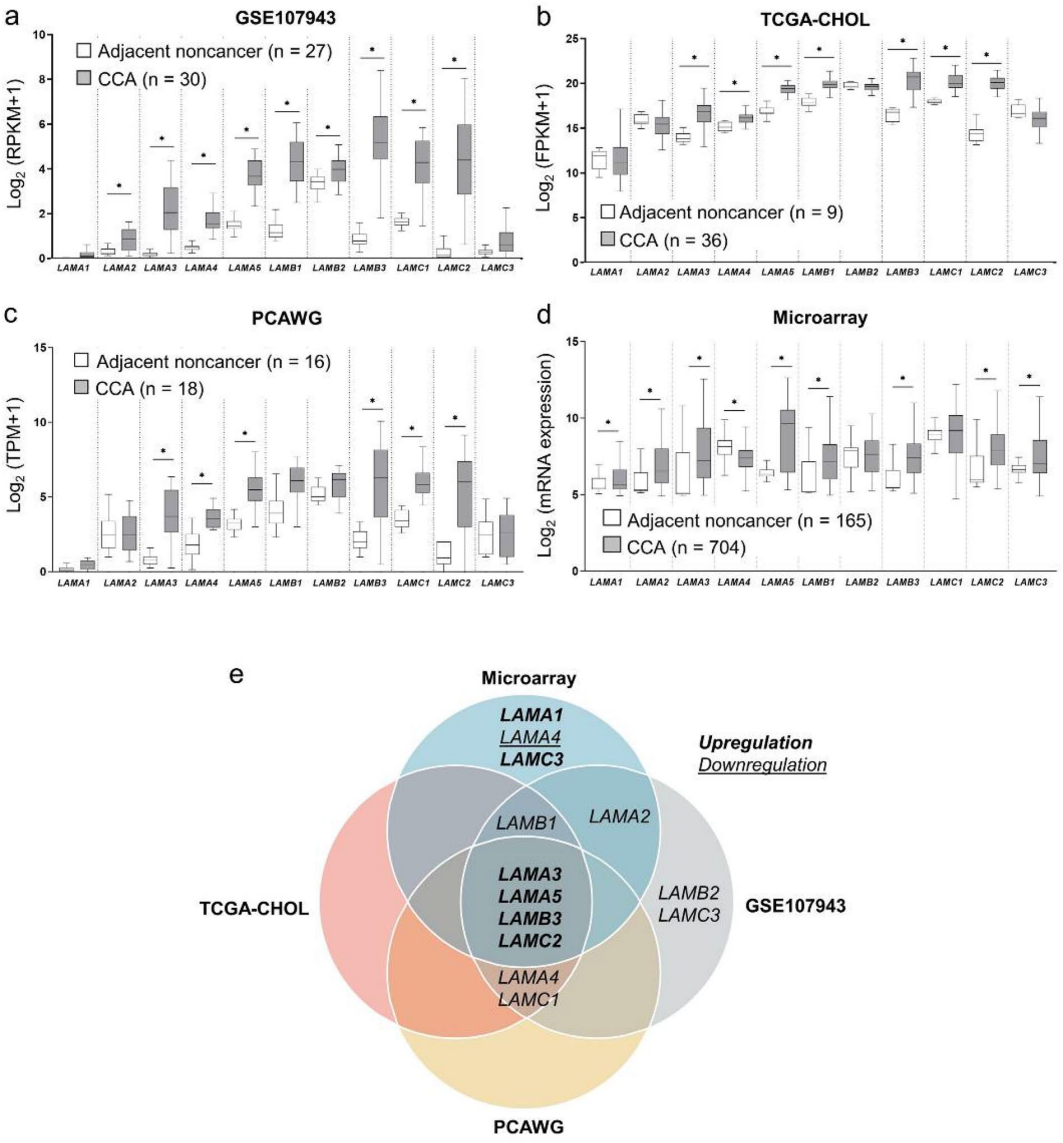
Gene expression profiling of laminin gene family in CCA through publicly available databases. Retrieved RNA-seq data from (**a**) GSE107943 dataset, (**b**) TCGA-CHOL cohorts and (**c**) PCAWG project, and (**d**) microarray data from ten integrated datasets. Graphs were created using Graphpad Prism. (**e**) Venn diagrams illustrating the common differentially upregulated genes in CCA shared by GSE107943, TGCA-CHOL, PCAWG and microarray. Statistically significant differences between groups were tested by Welch’s t-test. *P < 0.001.

To examine the differential expression of laminin gene family in CCA, the basal mRNA expression levels of LAMA1-5, LAMB1-3 and LAMC1-3 were obtained from three RNA-seq datasets retrieved from GEO, TCGA and PCAWG. The GSE107943 dataset obtained from GEO revealed nine overexpressed laminin genes in CCA compared to the adjacent noncancerous tissues, namely LAMA2, LAMA3, LAMA4, LAMA5, LAMB1, LAMB2, LAMB3, LAMC1, and LAMC2 (Fig. 1a). TCGA-CHOL cohort minimized the overexpressed genes from nine to seven, which included LAMA3, LAMA4, LAMA5, LAMB1, LAMB3, LAMC1 and LAMC2 (Fig. 1b), and analysis of the PCAWG data shared the six upregulated genes, except *LAMB1*, with the previous two datasets (Fig. 1c). Additionally, reprocessing of our previously collated microarray data revealed that out of 11, eight genes of laminin family, including LAMA1, LAMA2, LAMA3, LAMA5, LAMB1, LAMB3, LAMC2 and LAMC3 were significantly upregulated in CCA tissues when compared with the adjacent noncancerous tissues, and one gene, LAMA4, was downregulated (Fig. 1d). In sum, as demonstrated in Venn diagrams, LAMA3, LAMA5, LAMB3 and LAMC2 were the four genes which were concordantly overexpressed in four datasets (three RNA-seq and one microarray data) (Fig. 1e).

### Validation of the expression of laminin genes in CCA tissues and cell lines

**Figure 2:**
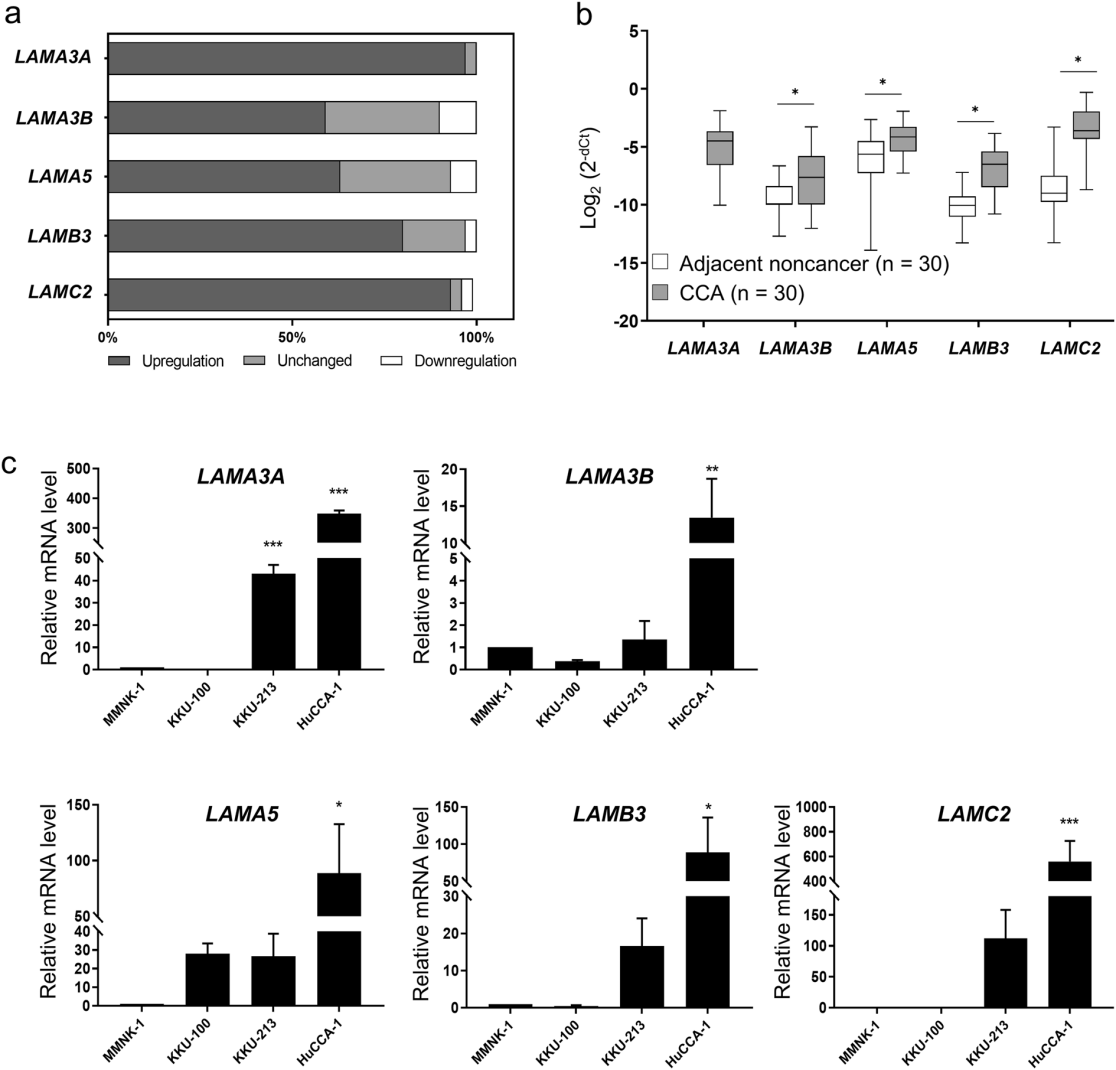
Validation of the expression of laminin genes in CCA tissues and cell lines using real-time RT-PCR. Relative mRNA levels of LAMA3A, LAMA3B, LAMA5, LAMB3 and LAMC2 in 30 pairs of CCA and their matched adjacent noncancerous tissues and in CCA cell lines were accessed using qPCR analysis normalized to *GAPDH*, and *18s rRNA,* respectively. (**a**) Percentage frequency of the cases with differential gene expression. Data were defined as upregulation and downregulation, according to FC ≥ 2 and FC ≤ 0.5, respectively. (**b**) The relative mRNA expression of CCA to their matched adjacent noncancerous tissues was evaluated using 2^−dCt^ formula and statistically analysed with Wilcoxon matched-pairs signed rank. (**c**) Relative mRNA levels of CCA cell lines to that of immortalized cholangiocyte MMNK-1 cells. Bar represents mean ± SEM of three independent experiments. Figures were created using Graphpad Prism. *P < 0.05, **P < 0.01, ***P < 0.001.

Here, we validated the upregulation of the four genes, namely LAMA3, LAMA5, LAMB3 and LAMC2, in 30 pairs of CCA and their matched adjacent noncancerous tissues as well as in CCA cell lines using real-time RT-PCR analysis. It is of note that LAMA3 has two transcript variants that are translated into proteins, LAMA3A and LAMA3B; nevertheless, only the truncated LAMA3 isoform is incorporated into the heterotrimeric laminins^14^. In tissues, the percentage of upregulated cases of the genes LAMA3A (29 cases; 97%), LAMA3B (17 cases; 56%), LAMA5 (19 cases; 66%), LAMB3 (24 cases; 80%) and LAMC2 (28 cases; 93%) outnumbered the combination of unchanged and downregulated cases (Fig. 2a). Mean FC of LAMA3B, LAMA5, LAMB3 and LAMC2 expression in CCA tissues compared to noncancerous counterpart were 3.5 (0.1–133 folds), 3.9 (0.3–216 folds), 9.2 (0.5–202 folds) and 37.7 (0.3–1,105 folds), respectively (Fig. 2b); however, there was no significant correlation between the expression levels and clinicopathology (Supplementary Table S1) as well as survival time of patients (Supplementary Fig. S1). Interestingly, LAMA3A relative expression was unable to be determined as its expression was too scarce to be detected in the majority of adjacent noncancerous tissues (27 out of 30) (Fig. 2b). The mRNA expression levels were further confirmed in CCA cell lines compared to immortal cholangiocytes MMNK‐1. HuCCA-1 significantly showed the highest level of all four laminin genes, and the enhanced LAMA3A was also demonstrated in KKU-213. Although there was no statistically significant difference in KKU-213 expression in other genes, the trend towards upregulation were observed (Fig. 2c). In view of the above results, we conducted an examination of LAMA3 expression in paraffin-embedded tissues obtained from 50 CCA patients through immunohistochemical staining. Nevertheless, we observed LAMA3 presence in 37 cases (74%) of CCA tissues, with its levels showing no significant correlation with either survival time or the clinicopathological aspects of the disease (Supplementary Fig. S2).

### Association of high LAMA3 expression with focal adhesion pathway

**Figure 3:**
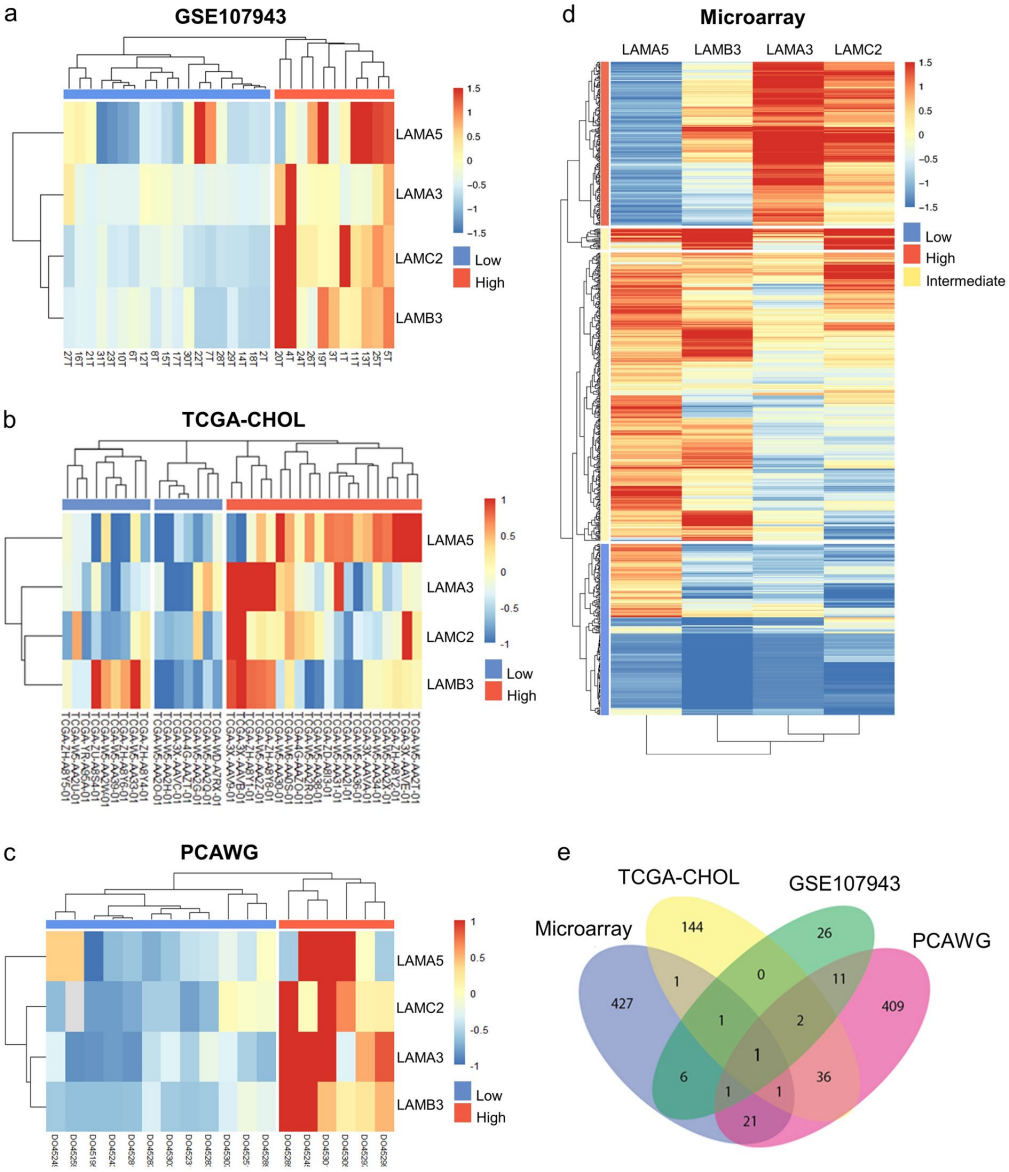
Identification of DEGs from hierarchal clustering analysis of laminin signature. Heatmap image of the four commonly upregulated laminin genes in (**a**) GSE107943, (**b**) TCGA-CHOL, (**c**) PCAWG and (**d**) microarray. (**e**) Venn diagram of DEGs listed from laminin signature in the four datasets. LAMA3 is the only DEG identified in all datasets. The DEGs screened based on |Log2FC|≥ 2 with adjusted *P*-value < 0.05. Figure were created using R Package.

Of all the datasets investigated, LAMA3, LAMA5, LAMB3 and LAMC2 were found to be commonly upregulated in cancer when compared to adjacent tissue samples as well as in CCA tissues and cell lines. Therefore, unsupervised hierarchical clustering analysis were performed using these four commonly upregulated genes which stratified the tumor tissue samples into high expression and low expression groups in all the cohorts (Fig. 3a–d). Further analysis to identify differential gene expression between the high and low expression groups in all the four different datasets investigated resulted in a total of 48 DEGs for GSE107943 dataset (Supplementary Table S2), 186 DEGs for the TCGA-CHOL (Supplementary Table S3), 482 DEGs for the PCAWG dataset (Supplementary Table S4) and 459 DEGs for the microarray datasets (Supplementary Table S5). The Venn diagram illustrating the intersection of the DEGs revealed LAMA3 as the sole gene to be differentially expressed amongst these DEGs in all datasets investigated (Fig. 3e).

**Figure 4:**
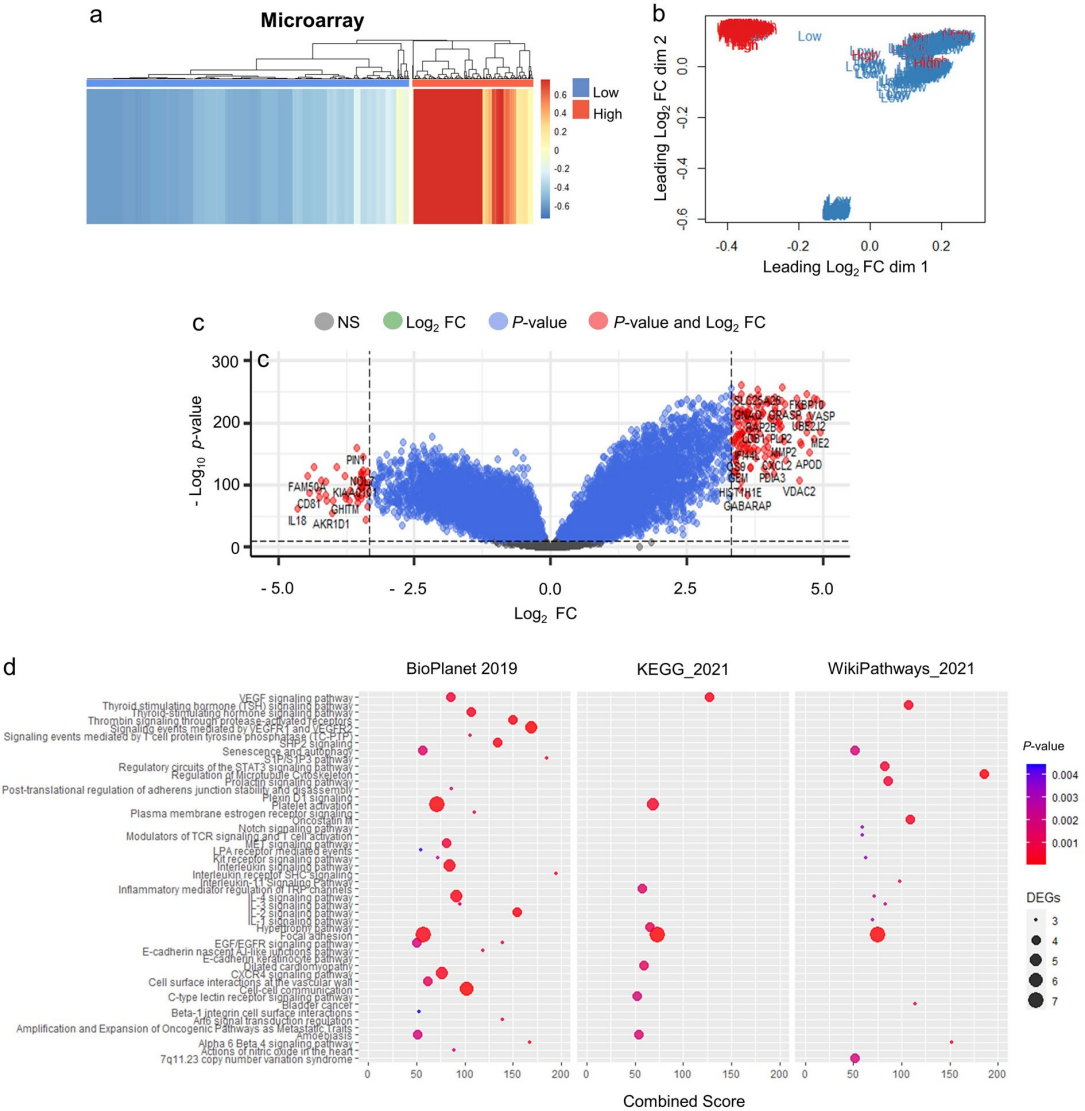
The DEGs and pathway enrichment analysis acquired unsupervised hierarchical clustering of LAMA3 stratified CCA sample of microarray dataset. (**a**) Heatmap image and (**b**) PCA plot indicating the separation low and high LAMA3 expression. (**c**) DEGs in high vs low LAMA3 expression group. Statistically significant DEGs cut-off are with the adjusted *P* < 0.05 and the Log2 FC of 3.32. (**d**) Pathway enrichment analysis of DEGs using EnrichR consolidated databases: BioPlanet, KEGG and Wikipathways. DEGs were considered at FC > 2 and* P* < 0.05. Enriched pathway were considered when combined score > 50.

We further restratified CCA samples according to LAMA3 expression level as a means to elucidate biological pathways specifically associated with this gene (Fig. 4a). Using PCA, we identified that the sample clusters of microarray in the low and high group were prominently distinguishable, and therefore was selected for this analysis. Transcriptomic changes and pathways affected were investigated (Fig. 4b). The result showed that 198 DEGs were found; with 155 upregulated and 43 downregulated as represented in the heatmap and the volcano plot (Fig. 4c and Supplementary Table S6), and these DEGs were concordantly enriched for ‘focal adhesion’ in three pathway databases consolidated in EnrichR, including BioPlanet, KEGG (Kyoto Encyclopedia of Genes and Genomes) and WikiPathways (Fig. 4d and Supplementary Table S7). The DEGs involved in focal adhesion pathways were upregulated, which included *ITGA7*, *PIK3R1*, *PPP1R12A*, *SRC*, *VASP* and *VEGFA*. The expression of some of these genes was explored in LAMA3 silencing KKU-213 using real time RT-PCR and we observed the statistically significantly decreased in *SRC* expression, but not in *ITGA7*, *PIK3R1* and *VASP* (Supplementary Fig. S3). Interestingly, several cancer progression-associated pathways, such as interleukin signalling, cell–cell communication and microtubule cytoskeleton regulation were enriched, suggesting the importance of LAMA3 in CCA malignancy (Fig. 4d). Together these results highlight the unique importance of LAMA3 in the laminin family in CCA.

### Effects of LAMA3 silencing on CCA cell proliferation, cell death and cell cycle progression

**Figure 5:**
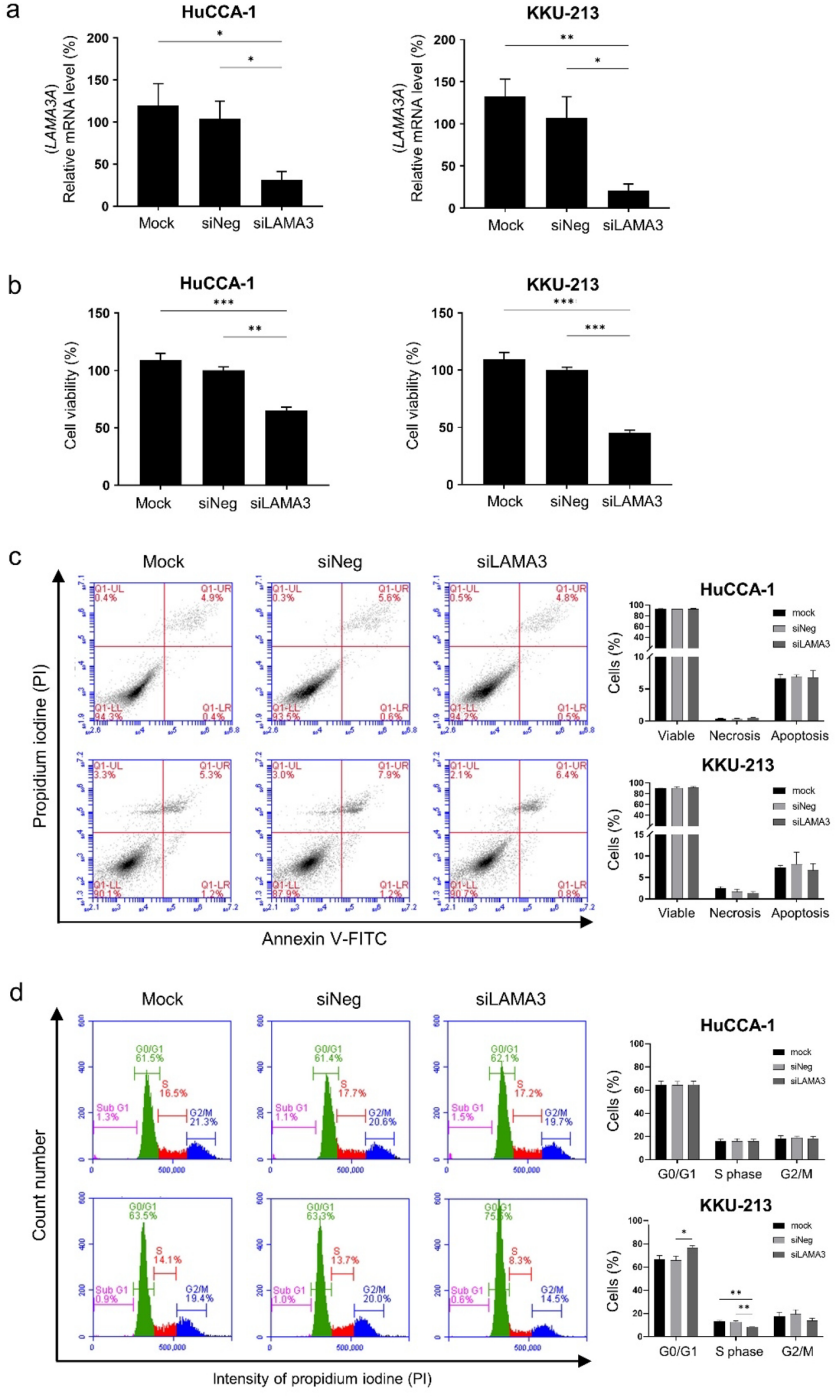
Effects of LAMA3 knockdown on CCA cell proliferation, cell death and cell cycle progression. (**a**) Efficiency of siLAMA3 on the reduction of LAMA3A expression in CCA compared with those of siNeg treatment at 48 h-post transfection. (**b)** Cell proliferation of CCA cell transfected with siLAMA3 determining at 72 h-post transfection using MTS assay. (**c)** Representative flow cytometry scatter plots and quantification for the percentage of apoptosis. (**d**) Representative area histograms and quantified bar graph for the distribution of cell cycle phases. Data present as mean ± SEM of siLAMA3 relative to siNeg from three independent experiments. Graphs were created using Graphpad Prism. Scatter plots and area histograms of flow cytometry were generated from The BD Accuri™ C6 Plus Flow Cytometer. *P < 0.05, **P < 0.01, ***P < 0.001.

Having recognized the significance of LAMA3 across the four datasets, the biological roles of LAMA3 on the progressive phenotypes of CCA were evaluated by the transient knockdown using siRNA targeting LAMA3 mRNA (siLAMA3). Compared with siNeg transfection, the siLAMA3 treatment effectively inhibited basal LAMA3A mRNA expression level of HuCCA-1 and KKU-213 by 73% and 86%, respectively (Fig. 5a), but barely affected expression of LAMA3B variant (Supplementary Fig. S4). In proliferation assay, cells were allowed to proliferate for 72 h before MTS assay. The data demonstrated that LAMA3 knocking down significantly reduced cell proliferation of both cell lines, substantially in KKU-213 with 55% decrease (Fig. 5b). To further understand the inhibitory effect of LAMA3 suppression on cell proliferation, we performed flow cytometry to investigate the induction of cell death and the distribution of specific phases of cell cycle. The results showed that suppression of LAMA3 did not affect cell death in both cell lines (Fig. 5c), but induced the G0/G1-phase arrest, resulting in the decreasing trend in S and G2/M phase entry of the cell cycle in KKU-213, compared with the control cells (Fig. 5d).

### Reduction of CCA cell adhesion and migration

**Figure 6:**
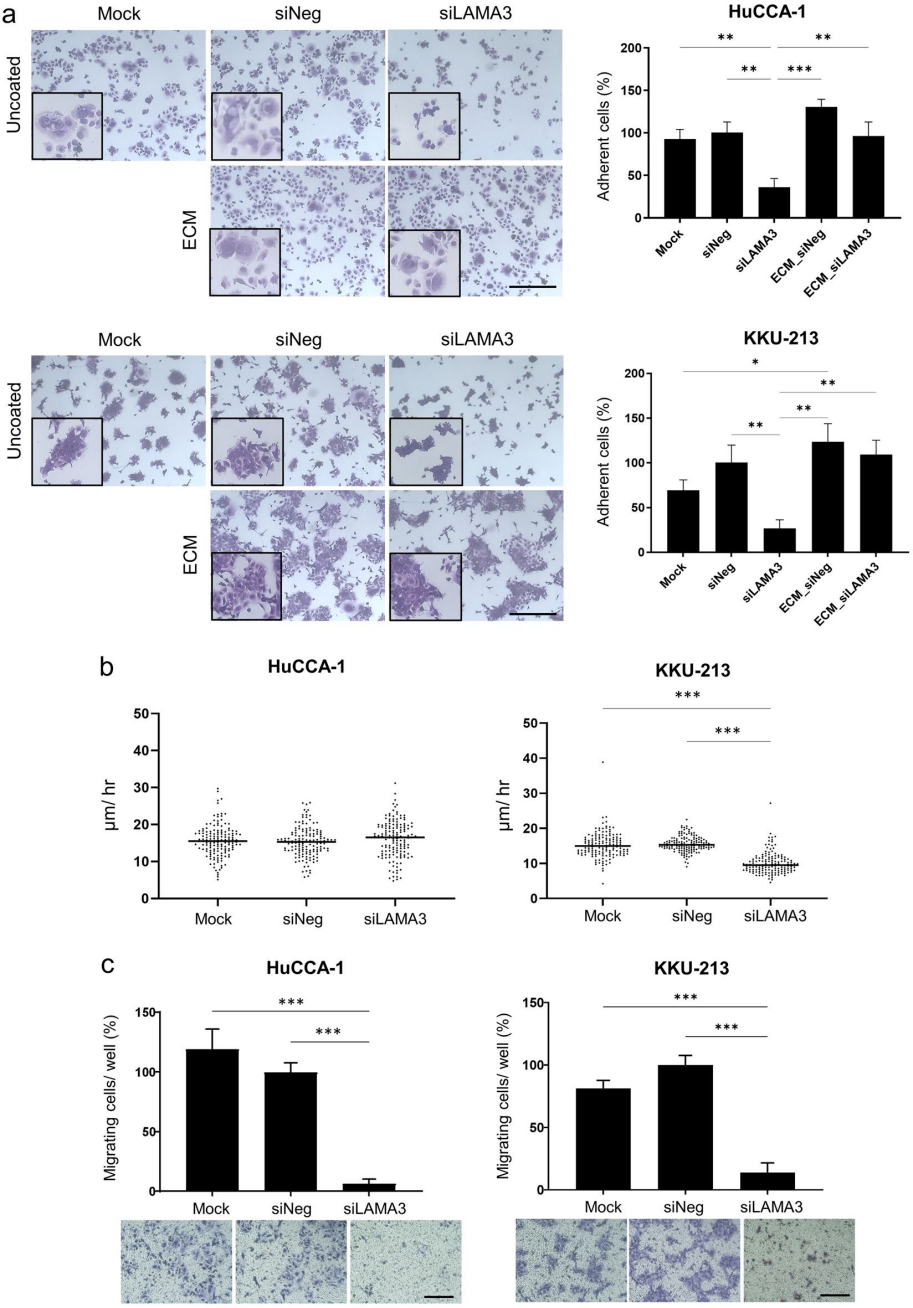
Reduction of CCA cell adhesion and migration in LAMA3-silenced cells. (**a)** Cell adherent area analyzed by Image J (right). Data present as mean ± SEM in percent relative to those of the siNeg from three independent experiments. Representative crystal violet staining of adherent cells under 10 × magnification and the inserts represent enlarge areas (left). Scale bar: 40 µm. ECM: mock-decellularized ECM-coated plate. (**b)** Migration speed of CCA cells collected by cell tracking in time-lapse every 15 min for 12 h. Data from one representative experiment were shown in dot plots, in which individual dots represent the average migration speed (µm/hour) per area (n = 150). (**c)** Cell migration examined by the transwell assay. Graphs were created using Graphpad Prism. *P < 0.05, **P < 0.01, ***P < 0.001.

Cell adhesion and cell migration are the critical steps in the invasive process of metastatic tumor cells, we assessed whether LAMA3 silencing dysregulated the adhesive and motility abilities of CCA cells. Forty-eight-hour-post transfected HuCCA-1 and KKU-213 cells were seeded and allowed to adhere on plate for 6 and 12 h, respectively, prior to the removal of non-adhered cells. Disrupting LAMA3 expression by siLAMA3 reduced adhesive ability of both CCA cells with only 36.0 ± 10.4% and 26.7 ± 9.7% of LAMA3-silenced HuCCA-1 and KKU-213 cells remained attached to the plate compared to the siNeg control (Fig. 6a); furthermore, LAMA3-silenced cells formed smaller cell spreading area as shown in the inserts of Fig. 6a. In addition, precoating of mock-derived decellularized ECM on the plate was able to restore the adhesive ability and the spreading area of both LAMA3-silenced cells to the comparable levels as those of the siNeg control cells (Fig. 6a). Tracking of the individual migrating cells using time-lapse video microscopy revealed that migration speed of LAMA3-silenced KKU-213 cells (10.1 ± 0.2 µm/hr) was significantly slower compared to that of the siNeg control cells (15.6 ± 0.2 µm/hr); however, the migration speed of LAMA3-silenced HuCCA-1 cells remained unchanged (Fig. 6b) (Supplementary Video S1 and S2). Therefore, we utilized in vitro transwell migration assay to investigate cell migration and found that in response to knocking-down of LAMA3, cell motility of HuCCA-1 and KKU-213 was declined by 93% and 86%, respectively, compared to that of siNeg treated cells (Fig. 6c), indicating the propensity roles of LAMA3 in promoting cell adhesion and migration.

### EMT status and cell morphology of LAMA3-silenced cells

**Figure 7:**
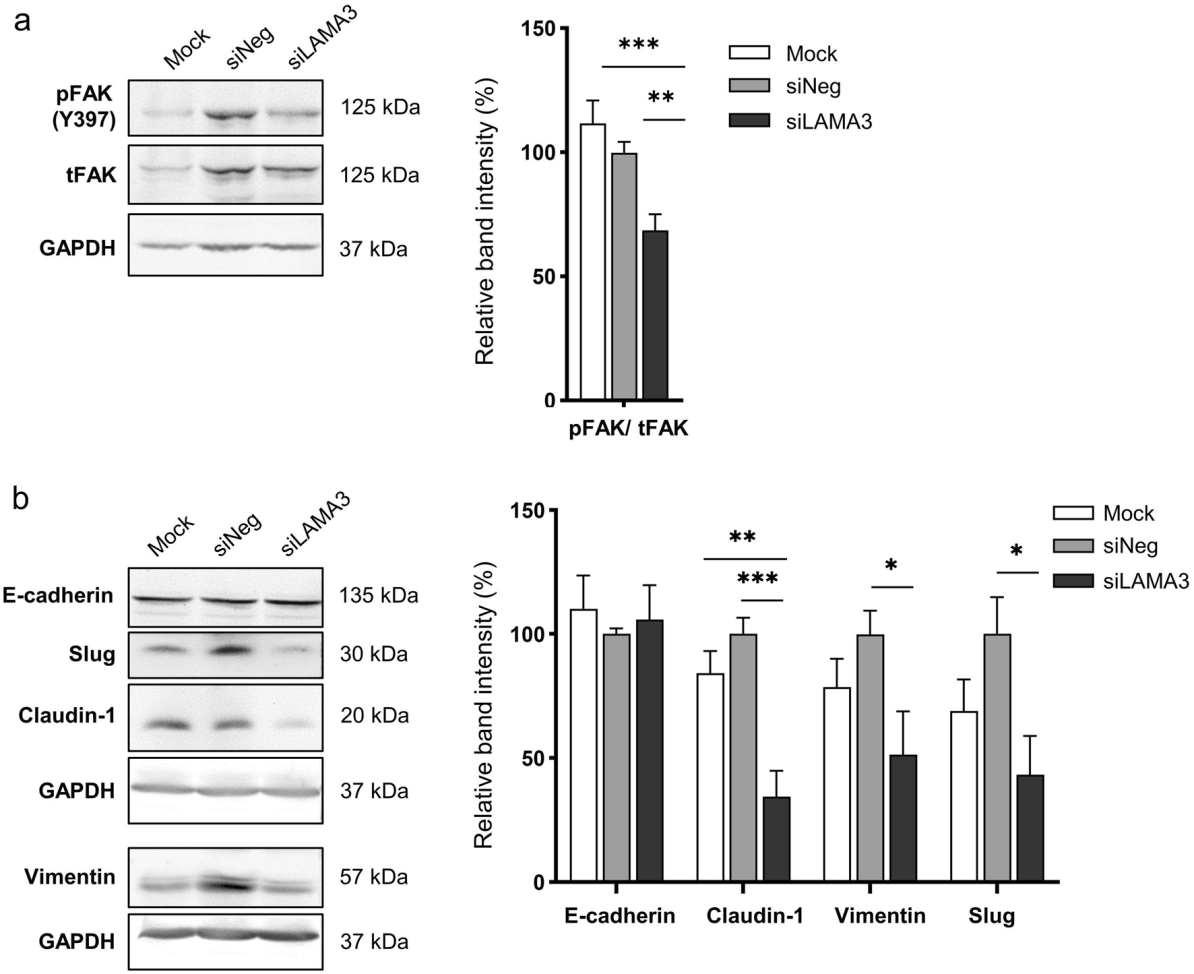
Representative western blotting illustrated the decrease in the pFAK/tFAK ratio and the inhibition of EMT markers expression in KKU-213 cells transfected with LAMA3 siRNA. Treated cells were reseeded for 24 h before protein extraction and GAPDH was used as the internal control. The cropped blots are used in the figure. The membranes were cut prior to exposure so that only the portion of gel containing the desired bands would be visualized. Full-length blots are presented in Supplementary Fig. S4. (**a**) Expression of FAK and its phosphorylation at Y397. (**b**) Expression of EMT-markers, including vimentin and Slug, and the epithelial markers, E-cadherin and claudin-1. Band intensity was showed as mean ± SEM in percent relative to those of the siNeg from at least four independent experiments. Graphs were created using Graphpad Prism. *P < 0.05, **P < 0.01, ***P < 0.001.

**Figure 8:**
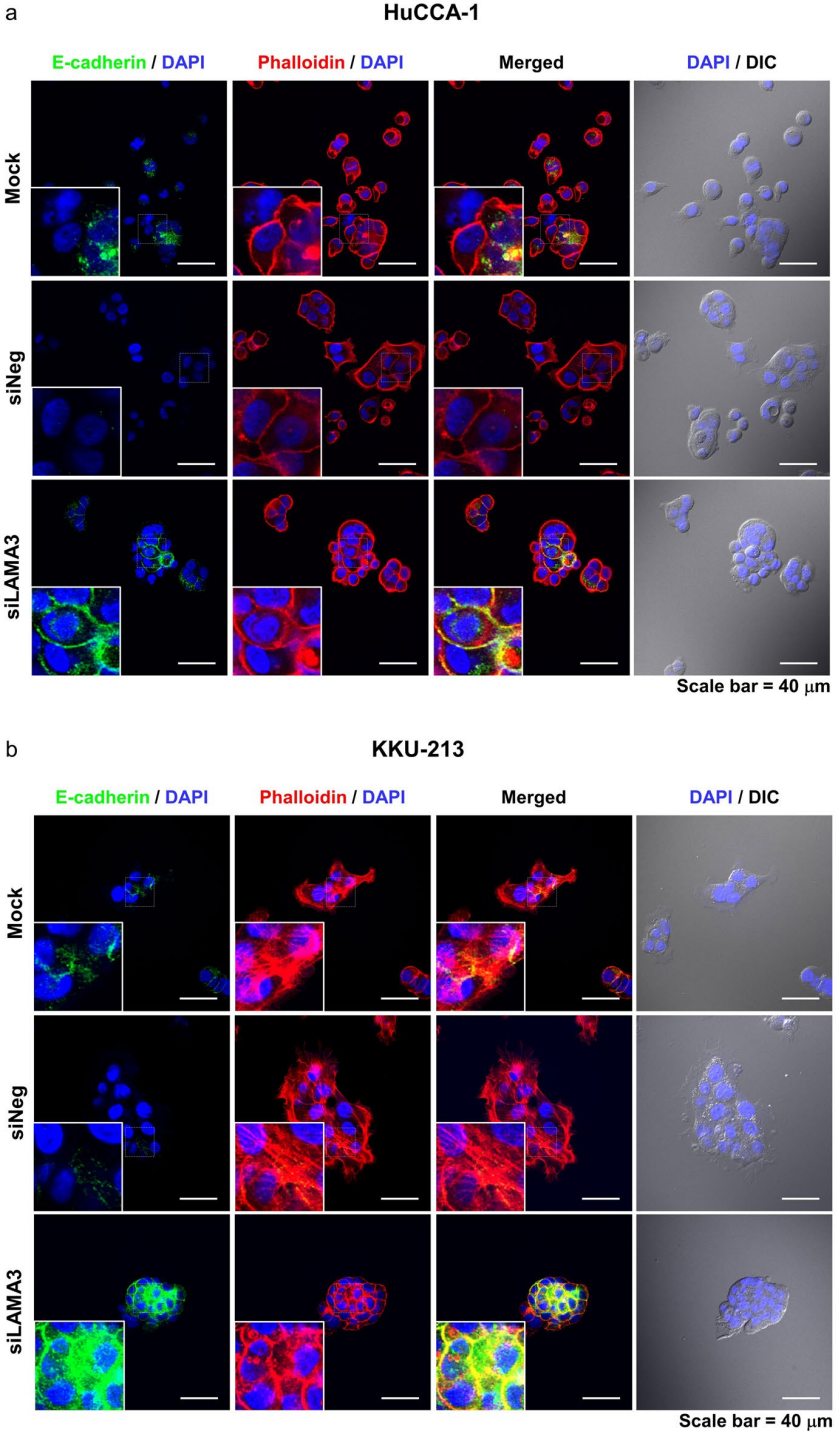

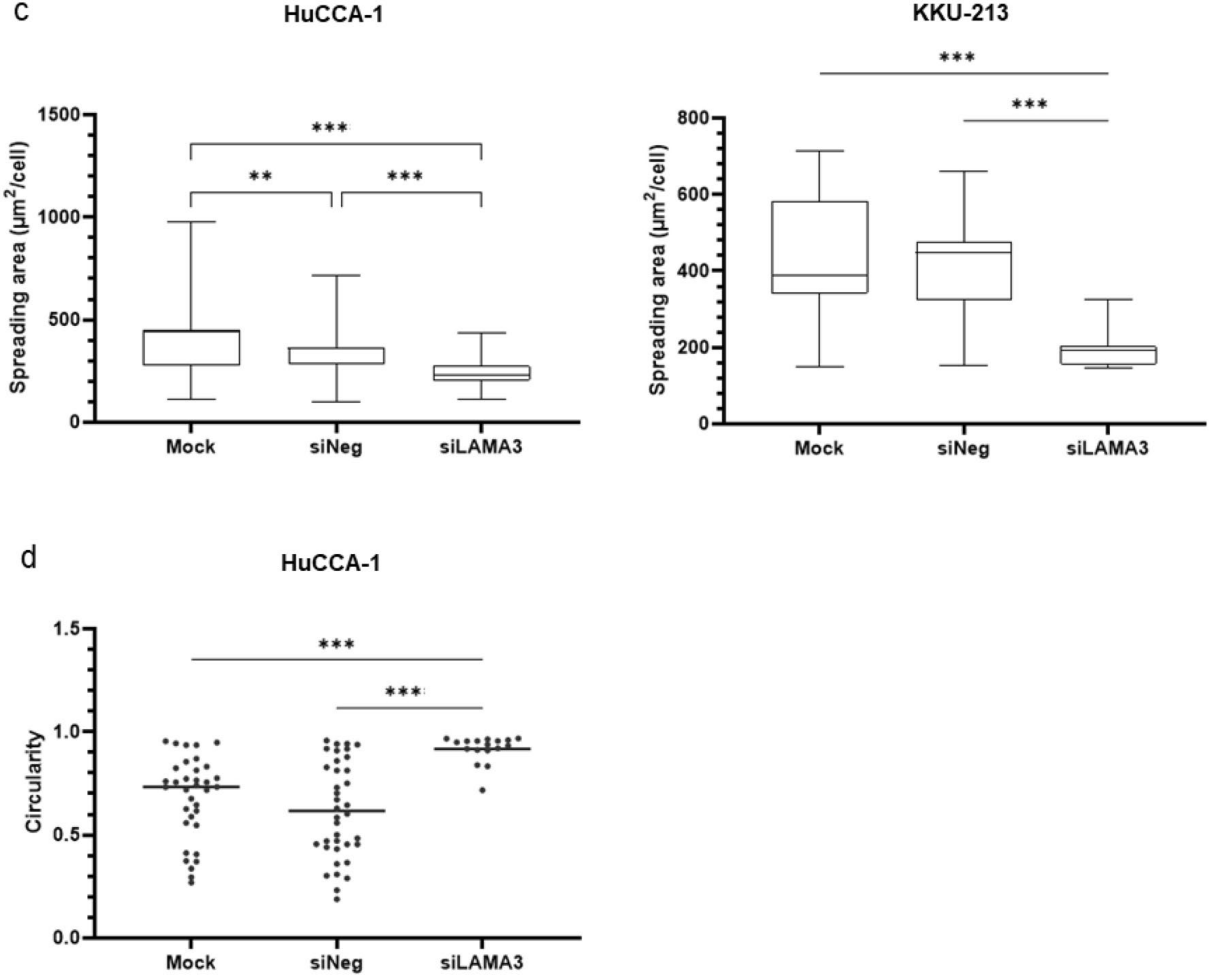
Alteration of E-cadherin and F-actin distribution and shrinkage of cell spreading area in CCA cells upon LAMA3 knockdown. Forty-eight-hr LAMA3-silenced cells were reseeded for 24 h before immunofluorescent staining. **(a**,**b)** Immunofluorescent images showed localization and distribution of E-cadherin protein (green), F-actin (using phalloidin staining, red), and nuclei (Hoechst staining, blue). **(a)** HuCCA-1; **(b)** KKU-213; scale bar: 40 µm. **(c)** The cell size in the unit area (μm^2^/cell) and **(d)** cell circularity was measured from phalloidin staining. Data from one representative experiment were shown in box plots. The value close to one indicates high circularity. (cell spreading: n > 40, circularity = n > 15). Images were captured from FV1000. **P < 0.01, ***P < 0.001.

Activation of focal adhesion kinase (FAK) through autophosphorylation at Tyr397 is involved in regulating EMT-mediated cancer progression and affects the expression of EMT-related proteins^15^. Hence, we determined the pFAK/tFAK ratio and the EMT status following LAMA3 knockdown, and it exhibited that inhibition of LAMA3 significantly decreased this ratio and suppressed the expression of the EMT-inducing transcription factor Slug, vimentin and claudin-1 but not significantly affected E-cadherin expression when compared with siNeg cells (Fig. 7, Supplementary Fig. S5). However, immunofluorescence staining demonstrated a significant increase in the localization of E-cadherin to the cell membrane in both CCA cells and particularly pronounced in the KKU-M213 cells after LAMA3 knockdown (Fig. 8a,b). Furthermore, phalloidin staining confirmed a reduction in cell spreading area by 27.8% and 56.2% in LAMA3-silenced HuCCA-1 and LAMA3-silenced KKU-213 cells, respectively, compared to siNeg-treated cells (Fig. 8c). Consistent with the decrease in cell spreading, we observed a significant increase in the circularity (close to one) of the HuCCA-1 cells after  LAMA3 silencing (Fig. 8d). However, due to the drastic loss in adhesive property of LAMA3-silenced KKU-213 cells, there were not enough single cells for measuring the circularity. Collectively, we clarified that LAMA3 knockdown could inhibit proliferation, adhesion and migration; induce the G0/G1-phase arrest; and regulate EMT in CCA cells.

## Discussion

Extensive literature has indicated that the aberrantly increased deposition of matrix proteins in CCA TME contributes to the enhanced malignant behaviors of the disease^16^. Hence, the better understanding of the associations between TME and the aggressiveness of CCA may be critical to improve the prognosis and therapy of CCA patients. We previously revealed that the exogenous laminin was the most potent inducer for CCA migration and adhesion^10^; however, the roles of endogenous laminins pertaining to CCA progression remain poorly understood. Here, bioinformatics analysis identified LAMA3, LAMA5, LAMB3, and LAMC2 as the commonly upregulated laminin subtypes in CCA and the unique overexpression of LAMA3 transcript was confirmed in Thai CCA patient tissues and cell lines. Furthermore, silencing of the candidate laminin subtype LAMA3 diminished CCA cell proliferation, adhesion and migration, together with reversing EMT progress.

The availability of huge amounts of transcriptome data has upgraded the knowledge in the cancer research field. The present meta-analysis selected the datasets based on the variety of transcriptomic techniques and ethnic heterogeneity, thus increasing the sample size and the reliability of results and adverting the difference of patients’ background. This may allow the development of more precise targeted strategies. In concordance with in silico analysis, real time RT-PCR validated the upregulation of LAMA3, LAMA5, LAMB3 and LAMC2 in Thai CCA cell lines and patient tissues in which LAMA3A was upregulated the most and in nearly all cases (29 out of 30), pointing out the potentiality to use LAMA3 detection as the diagnostic approach to distinguish CCA patients from normal cases. However, there was no correlation between the expression levels with clinicopathology and overall survival. This discrepancy might be clarified through large-scale clinical studies.

 The four upregulated laminin subtypes have been previously reported of their tumorigenic effects. LAMA5 is primarily important for the proliferation induction in cancer cells^17^ and also upregulated in CCA-associated fibroblasts^18^. LAMB3 is involved in invasive and metastatic behaviors of various cancers, especially pancreatic adenocarcinoma (PAAD) by modulating the PI3K (phosphoinositide 3-kinases)/ Akt signaling pathway^19^. The product of this gene was also distinctively detected in the secretome of HuCCA-1, but not that of hepatocarcinoma cell lines^20^. An aberrant overexpression of LAMC2 was usually associated with worse prognosis for patients with bile duct, bladder, colon and lung cancers^14^. In CCA, Pei Y. et al. demonstrated the link of highly expressed LAMC2 to the activation of the epidermal growth factor receptor (EGFR) signaling pathway and EMT which leads to cell invasion, migration and angiogenesis^21^. Unlike other dysregulated laminins, expression level and roles of LAMA3 across different tumors are paradoxical. Using GEPIA2 analysis, we here revealed that LAMA3 was upregulated in various types of cancers such as colon, kidney, liver, pancreas and rectum, but downregulated in some cancers, viz. breast, lung and skin (Supplementary Fig. S6)^22^. Highly expressed LAMA3A splice variant in head and neck squamous cell carcinoma is strongly associated with poor survival outcome^23^. On the other hand, loss of LAMA3 induces cell invasion and macrophage infiltration in cutaneous squamous cell carcinoma^24^. 

To identify gene expression pattern related to laminin expression signature, CCA tumors in each transcriptomic dataset were classified into low and high expression using hierarchical clustering analysis. Surprisingly, the analysis demonstrated that LAMA3 was the only DEG overlapped in all four datasets investigated. Further restratifing the tumor samples according to LAMA3 expression level could enrich the ‘focal adhesion’ in multiple pathway databases; however, in this part the enrichment pathway analysis was exclusively performed in microarray datasets owning to the clear separation between low and high populations (Fig. 4d, Supplementary Fig. S7). Focal adhesions are formed at the cell-ECM contact points to initiate signalling events important for a variety of biological processes like cell motility, cell proliferation, cell differentiation, regulation of gene expression and cell survival^7^. Interestingly, several cancer progression-associated pathways, such as interleukin signalling, cell–cell communication and microtubule cytoskeleton regulation were also enriched, suggesting the importance of LAMA3 in CCA malignancy. As the α3 chain generally harbors the binding sites for its cognate receptors and its importance has not been well understood in CCA^25^, these rationalized the functional investigation for LAMA3 on CCA progression.

In the current work, the roles of LAMA3 in CCA malignancy were investigated using two high LAMA3 expressing CCA cell lines, KKU-213 and HuCCA-1, established from Thai patients with intrahepatic CCA. Our findings demonstrated that LAMA3 knockdown hindered cell proliferation, adhesion and migration and EMT induction as shown by decreased expression of vimentin and Slug. The EMT-transducing transcription factor Slug activates vimentin expression and in turn represses E-cadherin^8^. However, we revealed that the reduction of Slug did not disturb E-cadherin expression and this result is in line with Huang’s work showing that silencing LAMA3 expression decreases vimentin but has no effect on E-cadherin expression in PAAD cell lines named PANC-1^26^. Claudin-1 has bifunctional roles: maintaining epithelial cell polarity and facilitating collective migration^27^. We found that silencing LAMA3 downregulated claudin-1 expression, suggesting its role in inducing collective migration in KKU-213 as observed in Supplementary Video S2.

Although the knockdown efficiency of siLAMA3 treatment was relatively close in the two cell lines, some LAMA3 silencing effects were remarkably different. For example, LAMA3 knockdown reduced migration speed of KKU-213 detected by time-lapse imaging, yet it did not affect those of HuCCA-1. Moreover, it decreased proliferation of KKU-213 (55%) more than that of HuCCA-1 (35%). These might account for several contributing factors. The most likely cause is that HuCCA-1 has a significant higher level of basal *LAMA3* mRNA so large amount of the predeposited LAMA3 secreted prior to knockdown (Supplementary Fig. S8) might play role in maintaining migration speed of LAMA3-silenced HuCCA-1. Consequently, we could observe the reduction in cell migration using transwell migration assay in which the cells were reseeded after knockdown, thus eliminating the LAMA3 predeposited effect. Secondly, their α3 chain may undergo different proteolytic post-translational modifications; for example, HuCCA-1, but not KKU-213, abundantly secretes plasminogen activator inhibitor type 1 (PAI-1), major inhibitor of plasminogen activator/plasmin system^28^. Finally, they might distinctively transduce intracellular signaling due to the difference in laminins’ cognate receptors; for instance, HuCCA-1 prominently expresses β4 integrin and its cooperative partner c-Met, while KKU-213 remarkably produces 36/67 kDa laminin receptor and ErbB2^10,29–32^.

The findings of this study have to be seen in light of some limitations. First, because not all the datasets we utilized in the current study provide histological information, our in silico results generalize the biological effects of LAMA3 in both intrahepatic CCA and extrahepatic CCA. Future research can investigate its connection with specific histological types of CCA. Second, we still have inadequate information about the clinicopathological data and demographic variables of the biological samples used in the in silico analysis. Third, it is worth noting that the number of matched paired-tissue samples from the same donors may yield a reliable result as paired-sample RNA-seq significantly enhances not only the sensitivity in the detection of differential expression but also the statistical power^33^, thus further research could be carried out to explore this point. Last but not least, our results should be carefully interpreted and applied due to the resemblance to PAAD in the abundance of ECM proteins, like LAMA3, LAMB3 and LAMC2^26,34,35^.

To our knowledge, this is the first report to reveal the expression signature of laminin gene family in CCA and the impacts of LAMA3 on promoting CCA cell proliferation, adhesion and migration. The analysis undertaken here has broadened the understanding of CCA TME and emphasizes the need to expand preclinical studies with appropriate animal models more closely mimicking CCA TME that would accurately predict stromal interaction with CCA cells. In addition, our findings suggest that LAMA3 may be the promising diagnostic marker and therapeutic target to overcome the desmoplastic nature of CCA.

## Materials and methods

### Clinical tissue specimens and cell culture

A total of 30 pairs of frozen CCA and matched adjacent noncancerous tissues were obtained from patients who had undergone hepatic resection at Srinagarind Hospital, Khon Kaen University, Thailand and the tissue specimens were confirmed for CCA by the pathologist. The study protocol was approved by The Human Research Ethics Committee, Khon Kaen University (HE571283) and exempted by Mahidol University Central Institutional Review Board (MU-CIRB 2019/098.2803). All methods were carried out in accordance with the World Medical Association Declaration of Helsinki and informed consent was obtained from all patients or legally authorized representatives included in the study.

Thai patient-derived CCA cell lines, KKU-100 (RRID:CVCL_3996)^36^ and KKU-213^37^ as well as the immortalized cholangiocyte cell line MMNK-1(RRID:CVCL_M266)^38^ were purchased from the Japanese Collection of Research Cell Bank (JCRB), and the intrahepatic CCA cell line HuCCA-1^39^ was generously provided by Professor Stitaya Sirisinha (Mahidol University, Bangkok, Thailand). Cell lines were cultured in HAM’s F-12 medium supplemented with 15 mM HEPES, 14 mM NaHCO_3_, 1% antibiotic–antimycotic solution (penicillin, streptomycin and amphotericin B) and 10% heat-inactivated fetal bovine serum (GIBCO) in a humidified incubator at 37°C with 5% CO_2_.

### Gene expression profiling of laminin gene family analyzed from public transcriptome databases

Baseline mRNA expression of laminin gene family (11 genes)—namely LAMA1-5, LAMB1-3 and LAMC1-3—in CCA tumor and adjacent noncancerous tissues was analyzed in three independent RNA-seq datasets, including GSE107943, The Cancer Genome Atlas (TCGA)^40^ and The International Cancer Genome project: Pan-Cancer Analysis of Whole Genomes (PCAWG; https://docs.icgc.org/pcawg)^41^. First, the RPKM (reads per kilobase of transcript per million reads mapped) data of GSE107943 dataset, containing 30 CCA samples with 27 matched normal liver tissues, was retrieved from GEO (www.ncbi.nlm.nih.gov/geo/), using the search terms "cholangiocarcinoma" AND "human" AND "RNA-seq". Second, the FKPM (fragments per kilobase of transcript per million reads mapped) data from TCGA-CHOL cohort containing 36 CCA tumor and nine match TCGA normal samples was obtained from cBioportal (www.cbioportal.org). Third, the TPM (transcript per million) data from PCAWG project was acquired from the ArrayExpress (www.ebi.ac.uk) using the accession number “E-MTAB-5423”, which contained 18 tumor tissues and 16 noncancerous adjacent tissues.

The transcriptomic profiles were further explored based on our previously collated microarray data obtaining from 10 independent cohorts (GSE132305, GSE2263, GSE26566, GSE32225, GSE32879, GSE35306, GSE5755, GSE66255, GSE76297 and GSE89749) from GEO database, which included 704 CCA tumor and 165 adjacent noncancerous tissues^42^. The combined datasets were undergone Log_2_(x + 1) transformation, where “x” is signal intensity. The differential expression of specified genes in CCA tumors and noncancerous tissues was compared using Welch’s* t*-test. *p*-value < 0.001 was considered differentially expressed genes.

### RNA isolation and quantitative real-time PCR (qRT-PCR)

RNA was extracted from liver tissues of CCA patients by coarsely homogenizing the tissue in TRIzol™ Reagent (Invitrogen) as previously described^43^. The RNA concentration and quality (260/280 ratio) were determined using a Nano Drop UV-spectrophotometer (Thermo Fisher Scientific). The RNA (2 µg) was reversely transcribed into cDNA employing random hexamer primers and HyperScript™ RT—Master mix (GeneAll Biotechnology). Real-time PCR analysis was performed in a 10-µl mixture containing cDNA (25 ng), 1X FastStart Universal SYBR Green Master cocktail (Roche Diagnostics) and specific primer pair (0.5 µM of each) using the CFX96 Touch System (Bio-Rad) under the following thermocycling reactions: 95 ˚C for 5 min; 40 cycles of 94˚C for 45 s; 55 ˚C for 30 s; and 72˚C for 30 s. The primers sequences were listed as follows: 18srRNA (5′-CCATCCAATCGGTAGTAGCG-3′, 5′-GTAACCCGTTGAACCCCATT-3′), *GAPDH* (5′-CACCAGGGCTGCTTTTAACTCTGG-3′, 5′-CCTTGACGGTGCCATGGAATTTGC-3′), *LAMA3A* (5′-GTTCACAGCAGCAAAGGGTG-3′, 5′-CAATTGCAGGGAACACACCG-3′), *LAMA3B* (5′-TAGACTTTGGAAGCACCTACTCA-3′, 5′-GTTTATCAAGGACACCACAACCT-3′), *LAMA5* (5′-TGCATCGAGATGGACACG-3′, 5′-GCTTCAGGAAGAAGAGCA-3′), *LAMB3* (5′-GGCTGCGACAAGGCATCA-3′, 5′-CACCG GGTAGCGATTACAGTA-3′), *LAMC2* (5′-CCAGGAGGGAAGTCTGTGATT-3′, 5′-TCTGTGCCGGTAAAAGCC AT-3′). Gene expression was quantified relative to the internal control gene GAPDH using the 2^−dCt^ method^44^. Fold-change (FC) for gene expression in CCA compared to matched adjacent noncancerous was calculated with the 2^−ddCt^ method, which defined Log_2_ FC > 1 as upregulation and <  − 1 as downregulation.

The RNA of cell lines (70–80% confluence) was extracted using a Blood/Cell Total RNA Mini Kit (Geneaid) following the manufacturer’s instruction. Relative mRNA levels of CCA cell lines to those of immortalized cholangiocyte MMNK-1 cells were quantified by 2^−ddCt^ method using 18S rRNA for normalization^44^. Three independent experiments were performed, each with triplicate assays.

### Differential gene expression analysis

Unsupervised hierarchical clustering was conducted to explore whether the filtered gene signature expression can be used to stratify CCA patients. Heatmap was drawn using the pheatmap package. Samples were then grouped according to the clusters corresponding to the low- and high-expression of the commonly upregulated laminin genes across all datasets. To assess the differences in transcriptomic profiles between samples with low and high expression of the filtered gene list, Principal Component Analysis (PCA) and differential gene expression analyses were conducted using the DESeq2 package (RRID:SCR_015687) for the RNA-seq datasets and limma package (RRID:SCR_010943) for the Microarray datasets in R version 4.0.2. *P*-values were determined by Wald statistics and an adjusted *P*-value (Q-value) to correct for multiple comparisons testing using the Benjamini–Hochberg method. DEGs were defined as genes with 2 × FC for GSE107943, TCGA-CHOL, PCAWG and 10 × FC for the microarray datasets with adjusted *P*-value < 0.05. Results were visualized using ‘Enhanced Volcano’ (RRID:SCR_018931) and ‘ggplot2’ (RRID:SCR_014601) packages. This analysis was also repeated using a re-stratification with just LAMA3 alone. Differentially expressed genes were then analyzed in iLincs SPIA analysis (www.ilincs.org; Accessed online on August 1st, 2022) using using EnrichR consolidated pathway analysis databases (https://maayanlab.cloud/Enrichr/; Accessed online on August 1st, 2022)^45–47^, namely BioPlanet, KEGG^48^ and WikiPathways for signaling pathway enrichment, which was sorted with DEGs > 2-FC, combined score > 50 and* P-*value< 0.05.

### Silencing of LAMA3 expression

Small interfering RNAs (siRNAs) were transfected into CCA cell lines using Lipofectamin RNAiMAX reagent (Thermo Fisher Scientific), according to the manufacturer’s instruction. Cells were seeded in a six-well plate at the density of 1.5 × 10^5^ cells/ well (HuCCA-1) and 2 × 10^5^ cells/ well (KKU-213), or in a 96-well plate at the density of 3,000 cells/ well (HuCCA-1) and 4,000 cells/ well (KKU-213) for 24 h prior to transfection with 15 nM siRNA targeting LAMA3 (siLAMA3) (sc-43145; Santa Cruz Biotechnology). AllStars Negative Control siRNA (1027280; Qiagen) was used as control (siNeg). Transfecting medium was replaced with fresh complete media at 24-h post-incubation. Forty-eight hour-post transfection, efficiency of siLAMA3 was evaluated using qRT-PCR and protein expression profile and cell biological phenotypes were assayed as described below.

### MTS assay

The effect of LAMA3 depletion on cell proliferation was examined using the MTS assay. After transfection, the cells were allowed to proliferate for 72 h prior to addition of MTS reagent (3-(4,5-dimethylthiazol-2-yl)-5-(3-carboxymethonyphenol)-2-(4-sulfophenyl)-2H-tetrazolium) (ab197010; Abcam) to each well and further incubation for four hours according to the manufacturer’s instructions. The absorbance at 490 nm was recorded using Multiskan Skyhigh Microplate Spectrophotometer (Thermo Fisher Scientific). The data were collected from three independent experiments each done in triplicate.

### Cell adhesion assay

After forty-eight hours of transfection, HuCCA-1 (1 × 10^4^ cells) and KKU-213 (1.5 × 10^4^ cells) in 100 µl were seeded in 96-well plates and incubated for 6 and 12 h, respectively. Non-adherent cells were removed. Adherent cells were washed twice with cold PBS, fixed with cold absolute methanol for 15 min, stained with 0.5% crystal violet for 10 min, washed with tap water and air-dried. Images were captured in four random fields under 4 × magnification and cell-adhesive area was measured using Image J 1.52h (RRID:SCR_003070). The mean adhesion area of siNeg from three independent experiments was set as the reference (100%), and the adhesion area for each experimental condition was calculated as a percentage relative to the siNeg mean. These percentages were then plotted as the average area for each condition. For decellularized ECM, HuCCA-1 (4,000 cells/ well) and KKU-213 (5,000 cells/ well) were seeded in 96-well plate to reach confluence at 48 h. Cells were incubated in warm 0.25% Trypsin–EDTA (GIBCO) for 8 min to complete cell detachment followed by aspiration of cells. Wells were rinsed twice and treated with culture medium supplemented with 10% FBS before usage.

### Time-lapse and transwell cell migration assays

CCA cell migration were evaluated using two assays: time-lapse and transwell cell migration. For time-lapse analysis, cells were seeded in the 96 well black polystyrene microplate (3603; Corning Incorporated) and transfected with siLAMA3 or siNeg for 48 h as previously described. Cells were stained with Hoechst dyes (1:500) (MedChemExpress) for 15 min and the cell motility was monitored using an Operetta High-Content Imaging System (PerkinElmer) with a 40 × objective lens. Fluorescence and bright field images were captured every 15 min for 12 h. Migration speed was calculated by nuclei tracking using the Columbus™ software (PerkinElmer).

For transwell migration assay, CCA cells suspension in serum-free media (10^5^ cells/200 µl) was added into the apical inserts of transwell (3422; Corning Incorporated) and placed into the lower compartment filled with 600 µl of complete medium. After incubation at 37°C in a humidified 5% CO_2_ incubator for 12 h, cells underneath the upper chamber membrane were fixed with 30% methanol for 30 min and stained with 0.5% crystal violet for 10 min. Migrating cells were counted from four visual fields observed under 20 × magnification of an inverted microscope.

### Flow cytometry

To investigate cell death, 48-h transfected CCA cells (5 × 10^5^ cells per well) were seeded into a 6-well plate for 24 h. Both adherent and non-adherent cells were harvested, wash twice with cold PBS and resuspended in Annexin V-binding buffer and stained with Annexin V (AV) (BioVision) and propidium iodide (PI) (Thermo Fisher Scientific) and then incubated at room temperature for 5 min in the dark. Cells (10^4^) were collected using BD Accuri C6 Plus flow cytometer (BD Biosciences) and the percentage of apoptotic cells was acquired from an addition of the percentage of early (AV + /PI-) and late (AV + /PI +) apoptotic cells.

Cell cycle progression was analyzed after forty-eight-hour transfection. Cells were harvested, fixed with cold 70% ethanol at 4 °C for 2 h and then washed twice with PBS. Cells were resuspended and incubated overnight in staining buffer (PBS with 100 µg/mL RNase A, 50 µg/mL PI and 0.1% Triton X-100) at 4 °C in the dark. Flow cytometry was used to determine cell cycle phases by measuring nuclear DNA content from the intensity of PI.

### Western blot analysis

Forty-eight-hour transfected KKU-213 cells were reseeded for 24 h, then proteins were extracted in lysis buffer containing 150 mM Tris–HCl pH 7.4, 150 mM NaCl, 0.1% SDS, 1% sodium deoxycholate, 1% Nonidet P-40, 50 mM NaF, 2 mM Na_3_VO_4_, 40 mM β-glycerophosphate, 1 mM dithiothreitol, and a protease inhibitor cocktail (Roche). Aliquots (25 μg) of protein were resolved by 10 or 15% sodium dodecyl sulfate polyacrylamide gel electrophoresis and electrotransferred onto nitrocellulose membrane. The membranes were then incubated with primary antibodies (viz. rabbit anti-pFAK (8556), rabbit anti-E-cadherin (3195), rabbit anti-vimentin (5714), rabbit anti-Slug (9585), and rabbit anti-claudin-1 (13255) antibodies (Cell Signaling); and rabbit anti-tFAK (sc-557; Santa Cruz Biotechnology) followed by goat anti-rabbit horseradish peroxidase (HRP)-conjugated IgG secondary antibodies (7074; Cell Signaling). GAPDH was utilized to normalize gel loading and detected with mouse anti-GAPDH antibody (sc-32233; Santa Cruz Biotechnology) followed by the goat anti-mouse HRP-conjugated IgG secondary antibody (7076; Cell Signaling). Clarity Western ECL reagent (BioRad) was employed to obtain chemiluminescent signals recorded by a G-Box Chemi XL system (Syngene).

### E-Cadherin, cytoskeleton staining and cell shape-descriptor analysis

48-h transfected CCA cells (1 × 10^4^ cells per well) were seeded for 24 h onto an 8-well chamber slide (30108; SPL Life Science). Cells then were fixed with 4% paraformaldehyde for 15 min, and permeabilized with 0.5% Triton X-100 for 20 min, and then subjected to a blocking step using 1% bovine serum albumin (BSA) in PBS at room temperature for 90 min. Then, the cells were probed with E-cadherin antibody (24E10) (3195; Cell Signaling) at a dilution of 1:1,600 in 1% BSA at 4° C overnight. The cells were probed with Alexa Fluor® 488 goat anti-rabbit antibody (A11011; Molecular Probes) at a dilution of 1:500 in 1% BSA for 1 h at room temperature. Cell nuclei and F-actin were stained for 1 h with Hoechst 33342, and phallodin-Alexa 546 (1:1000) (Thermo Scientific). Stained cells were captured with FV1000 (Olympus Corporation) under 60 × magnification.

To obtain the total area of cell spreading, the distribution area of F-actin was detected using phallodin staining and analyzed by Image J software using the ROI (Region of Interest) manager tool, in which the individual cells were selected manually. For a group of continually attached cells, the average distribution area was calculated by dividing the total distribution area by the number of nuclei stained with Hoechst. Particles along the image border and out of focus were excluded. Finally, the data of the F-actin spreading area from single cells and a group of cells were combined and visualized in the unit area of μm^2^/cell. The circularity of cells was investigated according to the following equations:

$$\mathrm{Circularity}=\frac{4\mathrm{ \pi A}}{{\mathrm{P}}^{2}}$$

where P is the perimeter and A is the cell area.

### Statistical analysis

All statistical analyses were performed using Graphpad Prism software version 7 (GraphPad Software, Inc., San Diego, California). Mean comparisons between groups were determined using Welch’s *t*-test, Wilcoxon matched-pairs signed rank or one-way ANOVA where appropriate. Data were expressed as mean ± standard error of mean (SEM) of at least three independent experiments and statistical significance was considered when *P*-value was < 0.05, unless indicated.

## Supplementary Information

Supplementary Information 1.

Supplementary Tables.

Supplementary Video S1.

Supplementary Video S2.

## Data Availability

The datasets analyzed in the current study are publicly available accordingly: GSE107943 (https://www.ncbi.nlm.nih.gov/geo/query/acc.cgi?acc=GSE107943); PCAWG (https://www.ebi.ac.uk/gxa/experiments/E-MTAB-5423/Downloads); TCGA-CHOL (https://portal.gdc.cancer.gov/projects/TCGA-CHOL); and collated microarray data were available in previous publication (10.3390/ph14090898)^41^. Processed data and R-scripts generated during the current study are provided in Figshare with the identifier 10.6084/m9.figshare.22332838.v1.

## References

[1] Banales, J. M. *et al.* Cholangiocarcinoma 2020: the next horizon in mechanisms and management. *Nat. Rev. Gastroenterol. Hepatol.* **17**, 557–588 (2020). 32606456 10.1038/s41575-020-0310-z PMC7447603

[2] Høgdall, D., Lewinska, M. & Andersen, J. B. Desmoplastic tumor microenvironment and immunotherapy in cholangiocarcinoma. *Trends Cancer* **4**, 239–255 (2018). 29506673 10.1016/j.trecan.2018.01.007

[3] Sirica, A. E. *et al.* Intrahepatic cholangiocarcinoma: Continuing challenges and translational advances. *Hepatology* **69**, 1803–1815 (2019). 30251463 10.1002/hep.30289 PMC6433548

[4] Fabris, L. *et al.* The tumour microenvironment and immune milieu of cholangiocarcinoma. *Liver Int.* **39**(Suppl 1), 63–78 (2019). 30907492 10.1111/liv.14098 PMC10878127

[5] Aumailley, M. *et al.* A simplified laminin nomenclature. *Matrix Biol.* **24**, 326–332 (2005). 15979864 10.1016/j.matbio.2005.05.006

[6] Belkin, A. & Stepp, M. Integrins as Receptors for Laminins.pdf. *Microsc. Res. Tech.* **51**, 280–3011 (2002). 10.1002/1097-0029(20001101)51:3<280::AID-JEMT7>3.0.CO;2-O 11054877

[7] Givant-Horwitz, V., Davidson, B. & Reich, R. Laminin-induced signaling in tumor cells. *Cancer Lett.* **223**, 1–10 (2005). 15890231 10.1016/j.canlet.2004.08.030

[8] Ribatti, D., Tamma, R. & Annese, T. Epithelial-mesenchymal transition in cancer: A historical overview. *Transl. Oncol.* **13**, 100773 (2020). 32334405 10.1016/j.tranon.2020.100773 PMC7182759

[9] Fabris, L., Cadamuro, M., Cagnin, S., Strazzabosco, M. & Gores, G. J. Liver matrix in benign and malignant biliary tract disease. *Semin. Liver Dis.* **40**, 282–297 (2020). 32162285 10.1055/s-0040-1705109

[10] Islam, K., Thummarati, P., Kaewkong, P., Sripa, B. & Suthiphongchai, T. Role of laminin and cognate receptors in cholangiocarcinoma cell migration. *Cell. Adhes. Migr.* **15**, 152–165 (2021). 10.1080/19336918.2021.1924422 PMC8143218 34014802

[11] Liao, C. H. *et al.* microRNA-329 suppresses epithelial-to-mesenchymal transition and lymph node metastasis in bile duct cancer by inhibiting laminin subunit beta 3. *J. Cell. Physiol.* **234**, 17786–17799 (2019). 30887508 10.1002/jcp.28404

[12] Aishima, S. *et al.* Biliary neoplasia with extensive intraductal spread associated with liver cirrhosis: A hitherto unreported variant of biliary intraepithelial neoplasia. *Hum. Pathol.* **39**, 939–947 (2008). 18430455 10.1016/j.humpath.2007.10.031

[13] Liu, W. *et al.* Aberrant expression of laminin γ2 correlates with poor prognosis and promotes invasion in extrahepatic cholangiocarcinoma. *J. Surg. Res.* **186**, 150–156 (2014). 24124977 10.1016/j.jss.2013.09.008

[14] Rousselle, P. & Scoazec, J. Y. Laminin 332 in cancer: When the extracellular matrix turns signals from cell anchorage to cell movement. *Semin. Cancer Biol.* (2019). 10.1016/j.semcancer.2019.09.026 31639412

[15] Xu, C. Y. *et al.* SphK1 modulates cell migration and EMT-related marker expression by regulating the expression of p-FAK in colorectal cancer cells. *Int. J. Mol. Med.* **39**, 1277–1284 (2017). 28405684 10.3892/ijmm.2017.2921

[16] Sirica, A. E. & Gores, G. J. Desmoplastic stroma and cholangiocarcinoma: Clinical implications and therapeutic targeting. *Hepatology (Baltimore, Md.)* **59**, 2397–2402 (2014). 10.1002/hep.26762 PMC3975806 24123296

[17] Pouliot, N. & Kusuma, N. Laminin-511: A multi-functional adhesion protein regulating cell migration, tumor invasion and metastasis. *Cell Adhes. Migr.* **7**, 142–149 (2013). 10.4161/cam.22125 PMC3544778 23076212

[18] Utispan, K. *et al.* Gene expression profiling of cholangiocarcinomaderivedfibroblast reveals alterations related totumor progression and indicates periostin as apoor prognostic marker. *Mol. Cancer* **9**, 13–32 (2010). 20096135 10.1186/1476-4598-9-13 PMC2841583

[19] Zhang, H. *et al.* LAMB3 mediates apoptotic, proliferative, invasive, and metastatic behaviors in pancreatic cancer by regulating the PI3K/Akt signaling pathway. *Cell Death Dis.* **10**, 230 (2019). 30850586 10.1038/s41419-019-1320-z PMC6408539

[20] Srisomsap, C. *et al.* Proteomic studies of cholangiocarcinoma and hepatocellular carcinoma cell secretomes. *J. Biomed. Biotechnol.* **2010**, 437143 (2010). 20069059 10.1155/2010/437143 PMC2801507

[21] Pei, Y. F., Liu, J., Cheng, J., Wu, W. D. & Liu, X. Q. Silencing of LAMC2 reverses epithelial-mesenchymal transition and inhibits angiogenesis in cholangiocarcinoma via inactivation of the epidermal growth factor receptor signaling pathway. *Am. J. Pathol.* **189**, 1637–1653 (2019). 31345467 10.1016/j.ajpath.2019.03.012

[22] Tang, Z., Kang, B., Li, C., Chen, T. & Zhang, Z. GEPIA2: An enhanced web server for large-scale expression profiling and interactive analysis. *Nucleic Acids Res.* **47**, W556-w560 (2019). 31114875 10.1093/nar/gkz430 PMC6602440

[23] Moller-Levet, C. S. *et al.* Exon array analysis of head and neck cancers identifies a hypoxia related splice variant of LAMA3 associated with a poor prognosis. *PLoS Comput. Biol.* **5**, e1000571 (2009). 19936049 10.1371/journal.pcbi.1000571 PMC2773424

[24] Caley, M. P. *et al.* Loss of the laminin subunit alpha-3 induces cell invasion and macrophage infiltration in cutaneous squamous cell carcinoma*. *Br. J. Dermatol.* **184**, 923–934 (2021). 32767748 10.1111/bjd.19471

[25] Aumailley, M. The laminin family. *Cell Adhes. Migr.* **7**, 48–55 (2013). 10.4161/cam.22826 PMC3544786 23263632

[26] Huang, C. & Chen, J. Laminin-332 mediates proliferation, apoptosis, invasion, migration and epithelial-to-mesenchymal transition in pancreatic ductal adenocarcinoma. *Mol. Med. Rep.* **23**, 11 (2021). 33179081 10.3892/mmr.2020.11649 PMC7673329

[27] Zhou, B., Moodie, A., Blanchard, A. A. A., Leygue, E. & Myal, Y. Claudin 1 in breast cancer: New insights. *J. Clin. Med.* **4**, 1960–1976 (2015). 26633531 10.3390/jcm4121952 PMC4693152

[28] Thummarati, P. *et al.* High level of urokinase plasminogen activator contributes to cholangiocarcinoma invasion and metastasis. *World J. Gastroenterol.* **18**, 244–250 (2012). 22294827 10.3748/wjg.v18.i3.244 PMC3261541

[29] Menakongka, A. & Suthiphongchai, T. Involvement of PI3K and ERK1/2 pathways in hepatocyte growth factor-induced cholangiocarcinoma cell invasion. *World J. Gastroenterol.* **16**, 713–722 (2010). 20135719 10.3748/wjg.v16.i6.713 PMC2817059

[30] Treekitkarnmongkol, W. & Suthiphongchai, T. High expression of ErbB2 contributes to cholangiocarcinoma cell invasion and proliferation through AKT/p70S6K. *World J. Gastroenterol.* **16**, 4047–4054 (2010). 20731018 10.3748/wjg.v16.i32.4047 PMC2928458

[31] Yang, X. H. *et al.* Disruption of laminin-integrin-CD151-focal adhesion kinase axis sensitizes breast cancer cells to ErbB2 antagonists. *Cancer Res.* **70**, 2256–2263 (2010). 20197472 10.1158/0008-5472.CAN-09-4032 PMC3310185

[32] Islam, K. *et al.* Bioinformatics and qPCR analyses of laminins’ cognate receptors in cholangiocarcinoma tissues reveal the integrin ITGB4 as a potential biomarker *Sci. Asia* **48**, 379–386 (2022).

[33] Chung, L. M. *et al.* Differential expression analysis for paired RNA-seq data. *BMC Bioinf.* **14**, 110 (2013). 10.1186/1471-2105-14-110 PMC3663822 23530607

[34] Islam, S. *et al.* ITGA2, LAMB3, and LAMC2 may be the potential therapeutic targets in pancreatic ductal adenocarcinoma: An integrated bioinformatics analysis. *Sci. Rep.* **11**, 10563 (2021). 34007003 10.1038/s41598-021-90077-x PMC8131351

[35] Yang, C. *et al.* Evaluation of the diagnostic ability of laminin gene family for pancreatic ductal adenocarcinoma. *Aging (Albany NY)* **11**, 3679–3703 (2019). 31182680 10.18632/aging.102007 PMC6594799

[36] Sripa, B. *et al.* Establishment and characterization of an opisthorchiasis-associated cholangiocarcinoma cell line (KKU-100). *World J. Gastroenterol.* **11**, 3392–3397 (2005). 15948244 10.3748/wjg.v11.i22.3392 PMC4315993

[37] Sripa, B. *et al.* Functional and genetic characterization of three cell lines derived from a single tumor of an Opisthorchis viverrini-associated cholangiocarcinoma patient. *Hum. Cell* **33**, 695–708 (2020). 32207095 10.1007/s13577-020-00334-w

[38] Maruyama, M. *et al.* Establishment of a highly differentiated immortalized human cholangiocyte cell line with SV40T and hTERT. *Transplantation* **77**, 446–451 (2004). 14966424 10.1097/01.TP.0000110292.73873.25

[39] Sirisinha, S. *et al.* Establishment and characterization of a cholangiocarcinoma cell line from a Thai patient with intrahepatic bile duct cancer. *Asian Pac. J. Allergy Immunol.* **9**, 153–157 (1991). 1666951

[40] Weinstein, J. N. *et al.* The cancer genome atlas pan-cancer analysis project. *Nat. Genet.* **45**, 1113–1120 (2013). 24071849 10.1038/ng.2764 PMC3919969

[41] Campbell, P. J. *et al.* Pan-cancer analysis of whole genomes. *Nature* **578**, 82–93 (2020). 32025007 10.1038/s41586-020-1969-6 PMC7025898

[42] Balasubramanian, B. *et al.* RTK25: A comprehensive molecular profiling strategy in cholangiocarcinoma using an integrated bioinformatics approach. *Pharmaceuticals* **14**, (2021). 10.3390/ph14090898 PMC8469883 34577598

[43] Puetkasichonpasutha, J., Namwat, N., Sa-Ngiamwibool, P., Titapun, A. & Suthiphongchai, T. Evaluation of p53 and its target gene expression as potential biomarkers of cholangiocarcinoma in thai patients. *Asian Pac. J. Cancer Prev.* **21**, 791–798 (2020). 32212809 10.31557/APJCP.2020.21.3.791 PMC7437311

[44] Yang, J., Yang, Q., Yu, S. & Zhang, X. Evaluation and validation of suitable reference genes for reverse transcription-quantitative polymerase chain reaction studies in cholangiocarcinoma patients and cell lines. *Oncol. Lett.* **11**, 2673–2681 (2016). 27073537 10.3892/ol.2016.4232 PMC4812119

[45] Chen, E.Y. *et al.* Enrichr: interactive and collaborative HTML5 gene list enrichment analysis tool. *BMC Bioinform.* **14**, 128 (2013). 10.1186/1471-2105-14-128 PMC3637064 23586463

[46] Kuleshov, M.V.* et al.* Enrichr: a comprehensive gene set enrichment analysis web server 2016 update. *Nucleic Acids Res.* **44**, W90–W97 (2016). 10.1093/nar/gkw377 PMC4987924 27141961

[47] Xie, Z. et al. Gene set knowledge discovery with Enrichr. *Curr. Protoc.* **1**, e90 (2021). 10.1002/cpz1.90 PMC8152575 33780170

[48] Kanehisa, M. & Goto, S. KEGG: Kyoto encyclopedia of genes and genomes. *Nucleic Acids Res.* **28**, 27–30 (2000). 10592173 10.1093/nar/28.1.27 PMC102409

