# Author Correction: Upregulated LAMA3 modulates proliferation, adhesion, migration and epithelial-to-mesenchymal transition of cholangiocarcinoma cells

**DOI:** 10.1038/s41598-024-72068-w

**Published:** 2024-09-17

**Authors:** Kittiya Islam, Brinda Balasubramanian, Simran Venkatraman, Parichut Thummarati, Janpen Tunganuntarat, Nut Phueakphud, Phongthon Kanjanasirirat, Tanawadee Khumpanied, Pornparn Kongpracha, Yingpinyapat Kittirat, Rutaiwan Tohtong, Tavan Janvilisri, Patompon Wongtrakoongate, Suparerk Borwornpinyo, Nisana Namwat, Tuangporn Suthiphongchai

**Affiliations:** 1 Department of Biochemistry, Faculty of Science, Mahidol University, Bangkok, 10400 Thailand; 2 Graduate Program in Molecular Medicine, Faculty of Science, Mahidol University, Bangkok, 10400 Thailand; 3 Department of Clinical Chemistry, Faculty of Allied Health Sciences, Chulalongkorn University, Bangkok, 10330 Thailand; 4 Excellent Center for Drug Discovery (ECDD), Faculty of Science, Mahidol University, Bangkok, 10400 Thailand; 5 Department of Biochemistry, Faculty of Medicine, Khon Kaen University, Khon Kaen, 40002 Thailand; 6 Cholangiocarcinoma Research Institute, Khon Kaen University, Khon Kaen, 40002 Thailand; 7 Present Address: Department of Medical Sciences, Regional Medical Sciences Center 2, Ministry of Public Health, Phitsanulok, 65000 Thailand; 8 Center for Neuroscience, Faculty of Science, Mahidol University, Bangkok, 10400 Thailand; 9 Department of Biotechnology, Faculty of Science, Mahidol University, Bangkok, 10400 Thailand; 10 Present Address: Department of Systems Biosciences and Computational Medicine, Faculty of Medicine, Khon Kaen University, Khon Kaen, 40002 Thailand

Correction to *Scientific Reports* 10.1038/s41598-023-48798-8, published online 18 December 2023

The original version of this Article contained an error in the Methods section in which “sc-43145” was incorrectly given as “sc-35789”.

Under the subheading ‘Silencing of LAMA3 expression’,

“Cells were seeded in a six-well plate at the density of 1.5 × 10^5^ cells/ well (HuCCA-1) and 2 × 10^5^ cells/ well (KKU-213), or in a 96-well plate at the density of 3000 cells/ well (HuCCA-1) and 4000 cells/ well (KKU-213) for 24 h prior to transfection with 15 nM siRNA targeting LAMA3 (siLAMA3) (sc-35789; Santa Cruz Biotechnology).”

now reads:

“Cells were seeded in a six-well plate at the density of 1.5 × 10^5^ cells/ well (HuCCA-1) and 2 × 10^5^ cells/ well (KKU-213), or in a 96-well plate at the density of 3000 cells/ well (HuCCA-1) and 4000 cells/ well (KKU-213) for 24 h prior to transfection with 15 nM siRNA targeting LAMA3 (siLAMA3) (sc-43145; Santa Cruz Biotechnology).”

The original Article has been corrected.

