# Supplementary material for: Upregulated LAMA3 modulates proliferation, adhesion, migration and epithelial‑to‑mesenchymal transition of cholangiocarcinoma cells
Source: Sci Rep. 2023 Dec 18;13:22598. doi: 10.1038/s41598-023-48798-8 (PMC10730521; doi:10.1038/s41598-023-48798-8)
Supplement: Supplementary file 1 — Supplementary Information 1. [file 41598_2023_48798_MOESM1_ESM.pdf]

Upregulated LAMA3 modulates proliferation, adhesion, migration and epithelial-to-mesenchymal transition of cholangiocarcinoma cells

Kittiya Islam[1], Brinda Balasubramanian[1], Simran Venkatraman[2], Parichut Thummarati[3], Janpen Tunganuntarat[1], Nut Phueakphud[1], Phongthon Kanjanasirirat[4], Tanawadee Khumpanied[4], Pornparn Kongpracha[1], Yingpinyapat Kittirat[5,6], Rutaiwan Tohtong[1], Tavan Janvilisri[1], Patompon Wongtrakoongate[1,7], Suparerk Borwornpinyo[4,8], Nisana Namwat[5,6], Tuangporn Suthiphongchai[1*]

[1] Department of Biochemistry, Faculty of Science, Mahidol University, Bangkok 10400, Thailand

[2] Graduate Program in Molecular Medicine, Faculty of Science, Mahidol University, Bangkok 10400, Thailand

[3] Department of Clinical Chemistry, Faculty of Allied Health Sciences, Chulalongkorn University, Bangkok 10330, Thailand

[4] Excellent center for drug discovery (ECDD), Faculty of Science, Mahidol University, Bangkok 10400, Thailand

[5] Department of Biochemistry, Faculty of Medicine, Khon Kaen University, Khon Kaen 40002, Thailand.

[6] Cholangiocarcinoma Research Institute, Khon Kaen University, Khon Kaen 40002, Thailand.

[7] Center for Neuroscience, Faculty of Science, Mahidol University, Bangkok 10400, Thailand.

[8] Department of Biotechnology, Faculty of Science, Mahidol University, Bangkok 10400, Thailand.

* Corresponding author, e-mail: tuangporn.sut@mahidol.ac.th

Supplementary Figure S1. Survival analysis of CCA patients with differentially expressed LAMA3A, LAMA3B, LAMA5, LAMB3, and LAMC2 genes, using Kaplan-Meier method.

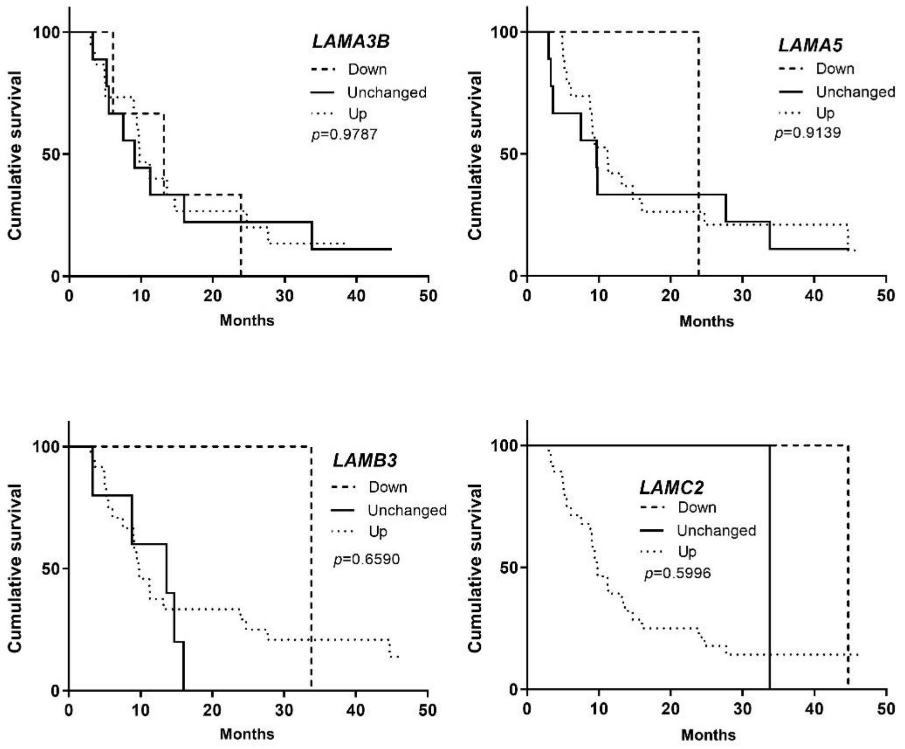

Supplementary Figure S1. Survival analysis of CCA patients with differentially expressed LAMA3A, LAMA3B, LAMA5, LAMB3, and LAMC2 genes, using Kaplan-Meier method.

Supplementary Figure S2. Immunohistochemical staining for LAMA3 in 50 CCA tissues.

a

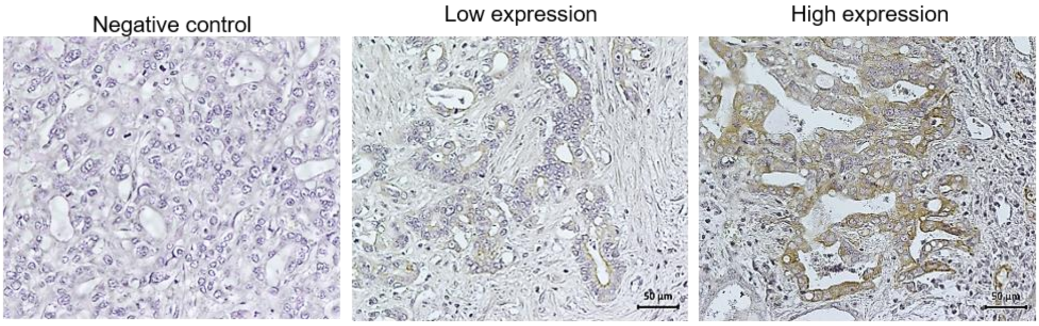

b

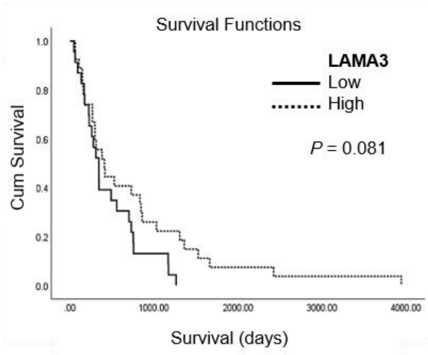

c

Factors	No. of patients (n=50)	LAMA3 expression		
		Low	High	*P*-value
**Sex**				
Female	20	12	8	0.091
Male	30	11	19	
**Age**				
< 58	25	12	13	0.500
≥ 58	25	11	14	
**Histological types**				
Non-papillary	31	13	19	0.328
Papillary	19	10	9	
**Metastasis**				
Non-metastasis	27	10	17	0.137
Metastasis	23	13	10	

Supplementary Figure S2. Immunohistochemical staining for LAMA3 in 50 CCA tissues. LAMA3 was detected on the paraffin-embedded CCA tissue sections using standard immunohistochemistry protocols. After blocking non-specific bind with 10% skim milk in PBS for 1 hr, sections were probed with rabbit polyclonal anti-LAMA3 antibody (HPA009309; Sigma) for 1 h at room temperature and overnight incubation at 4 °C, followed by incubation with anti-rabbit secondary antibody (G21234; Invitrogen) for 3 h. The color was developed with a 3, 3′ diaminobenzidine tetrahydrochloride (DAB) substrate kit (Vector Laboratories). The sections were counterstained with hematoxylin and examined under a light microscope (Eclipse, Ni-U, Nikon Instruments Inc). The LAMA3 expression was evaluated by considering both the frequency and intensity of staining. The frequency of staining was assessed semiquantitatively using the following criteria: no positive cells (0%), +1 for 1–25%, +2 for 26–50%, and +3 for >50%. The staining intensity was graded as mild (1), moderate (2), or intense (3). The final immunohistochemical score was computed by multiplying the intensity score with the frequency score. The median value, obtained from grading all patient scores, was used as a cut-off. Patients with scores below the median were categorized as having low expression, while those with scores equal to or surpassing the median were categorized as having high expression. a) Immunohistochemical staining LAMA3 in CCA tissues. b) Kaplan-Meier plots for LAMA3 expression. c) Correlation of LAMA3 expression with the clinicopathology.

Namwat N., *et al.* Downregulation of reversion-inducing-cysteine-rich protein with Kazal motifs (RECK) is associated with enhanced expression of matrix metalloproteinases and cholangiocarcinoma metastases. *J Gastroenterol*. **46**, 664-675 (2011).

Supplementary Figure S3. Effect of LAMA3 knockdown on *ITGA7*, *PIK3R1*, *SRC* and *VASP* expression in KKU-213

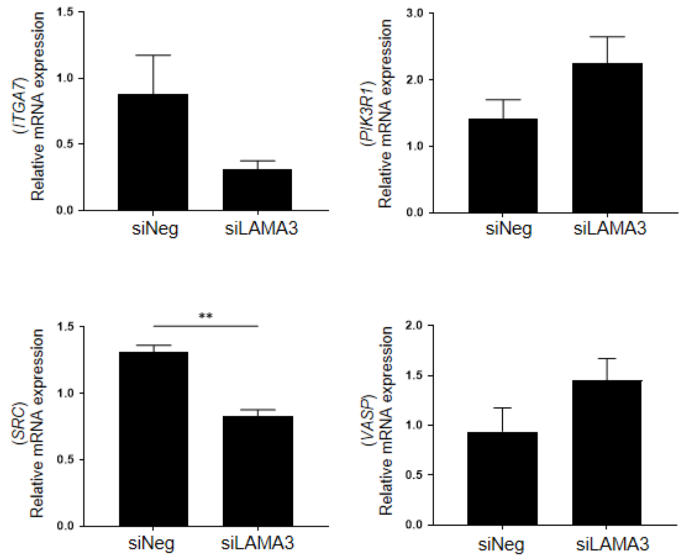

Supplementary Figure S3. Effect of LAMA3 knockdown on *ITGA7*, *PIK3R1*, *SRC* and *VASP* expression in KKU-213. Four-eight hour-post siLAMA3 transfection, KKU-213 cells were reseeded for 24 hours and harvested for RNA extraction. Data present as mean ± SEM relative to mock from three independent experiments and compared by *t*-test. **$P < 0.01$.

Supplementary Figure S4. Efficiency of siLAMA3 knockdown on the expression of LAMA3B variant relatively quantified by qRT-PCR.

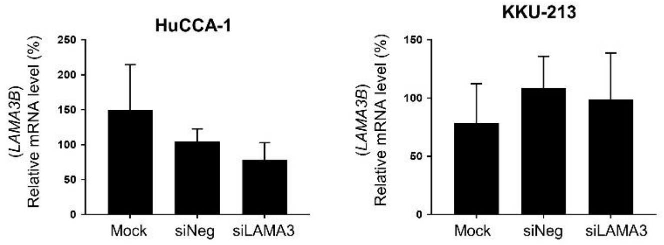

Supplementary Figure S4. Efficiency of siLAMA3 knockdown on the expression of LAMA3B variant relatively quantified by qRT-PCR. Efficiency of siLAMA3 on the reduction of LAMA3B expression in CCA compared with those of siNeg treatment at 48 hour-post transfection. Data present as mean ± SEM of siLAMA3 relative to siNeg from three independent experiments.

Supplementary Figure S5. Original full western blot exposures of the blots shown in the manuscript

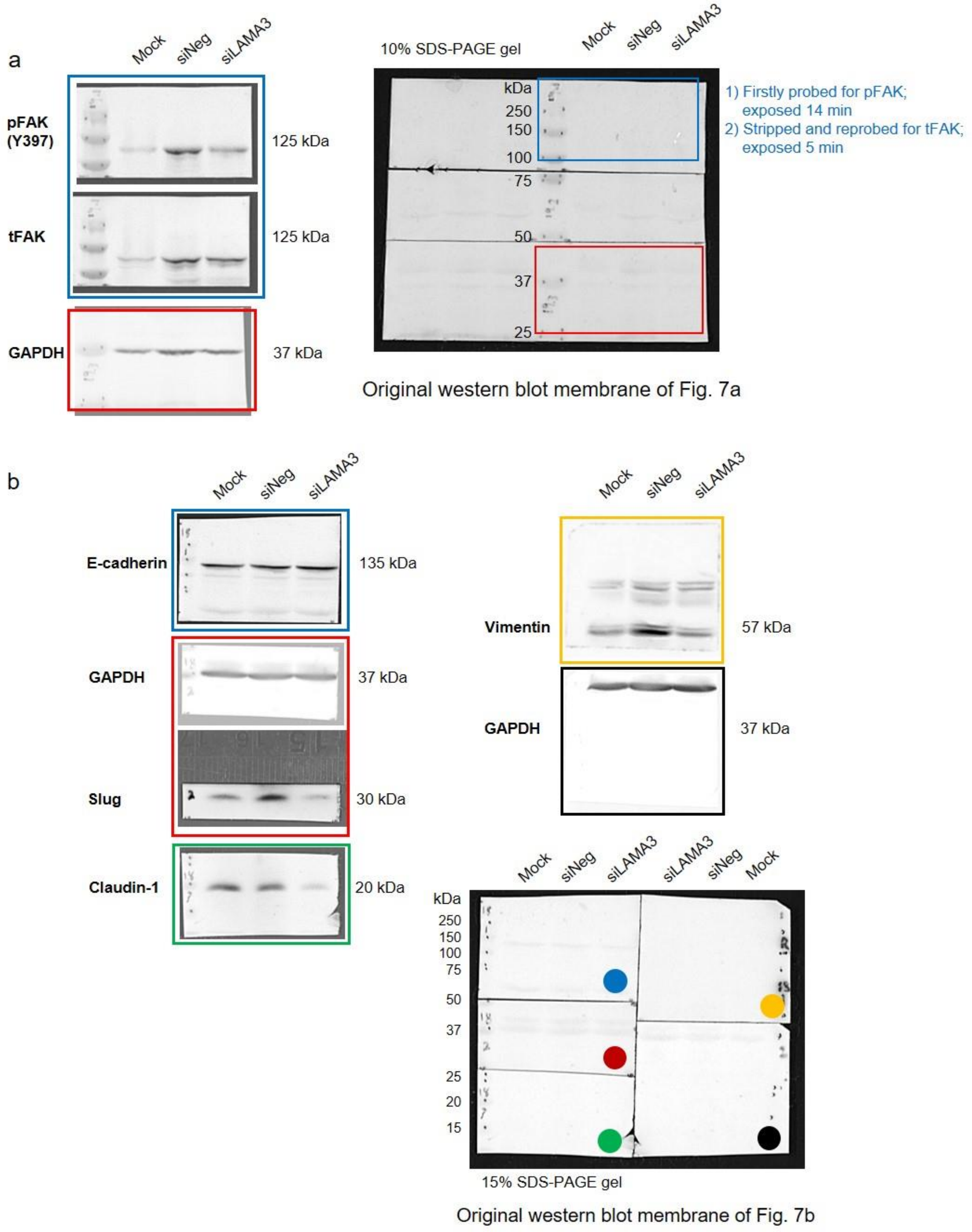

Supplementary Figure S5. (**a**) The cropped areas used in Fig.7a are shown in the frames of the original full western blot membrane (right). (**b**) For the western blot of EMT-markers presented in Fig.7b, the original full membrane (right, lower) was cut and shown with the matched colors to cropped area in the frames (left).

Supplementary Figure S6. The GEPIA2 analysis of TCGA data of LAMA3 expression across various cancers

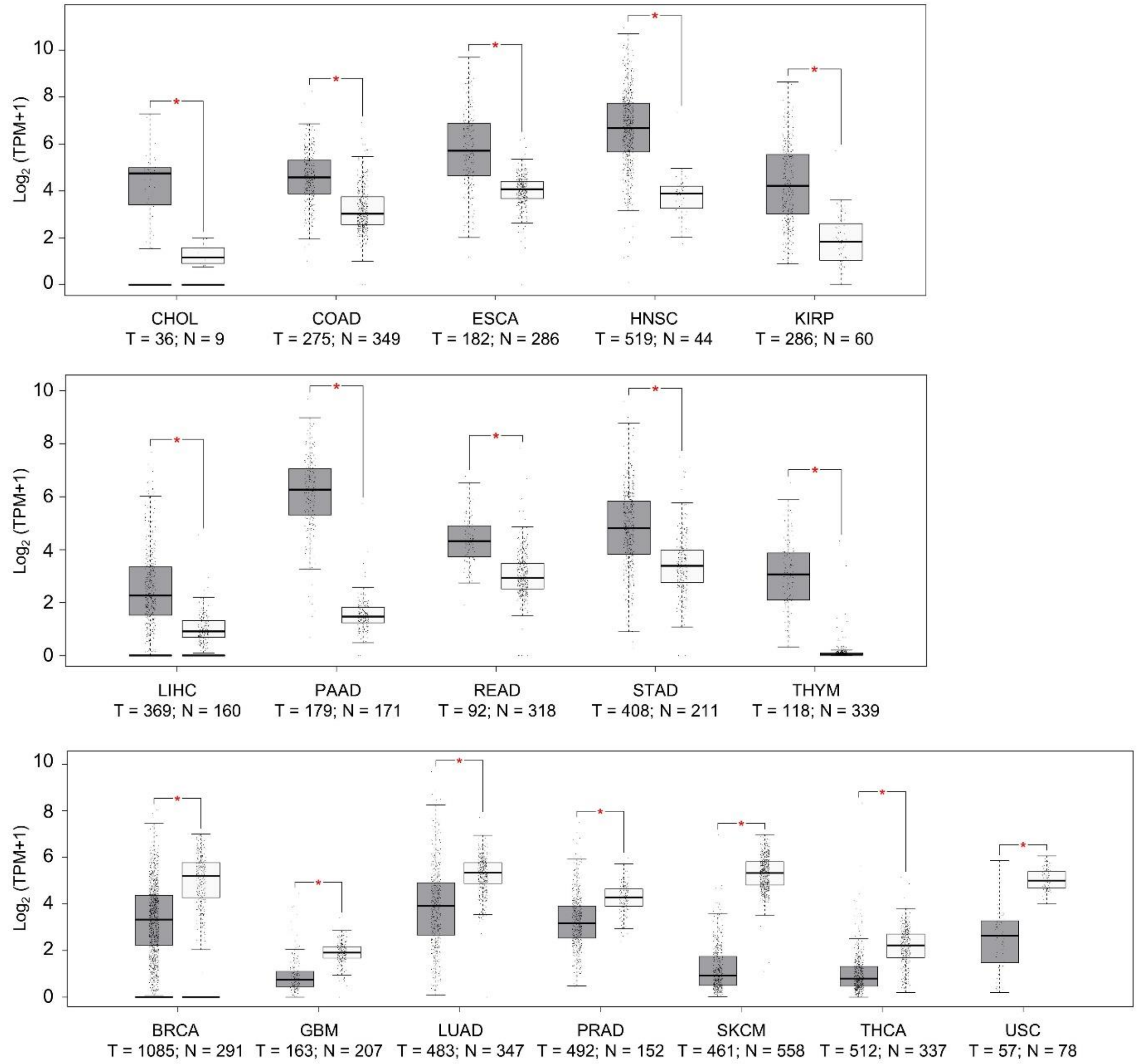

Supplementary Figure S6. The GEPIA2 analysis of TCGA data of LAMA3 expression across various cancers. BRCA: Breast invasive carcinoma, CHOL: Cholangiocarcinoma, COAD: Colon adenocarcinoma, ESCA: Esophageal carcinoma, GBM: Glioblastoma multiforme, HNSC: Head and neck squamous cell carcinoma, KIRP: Kidney renal papillary cell carcinoma, LIHC: Liver hepatocellular carcinoma, LUAD: Lung adenocarcinoma, PAAD: Pancreatic adenocarcinoma, READ: Rectum adenocarcinoma, PRAD: Prostate adenocarcinoma, SKCM: Skin Cutaneous Melanoma, STAD: Stomach adenocarcinoma, THCA: Thyroid carcinoma, THYM: Thymoma, UCS: Uterine Carcinosarcoma

Supplementary Figure S7. Volcano plot of DEGs and PCA plot between low vs high LAMA3 group in the datasets

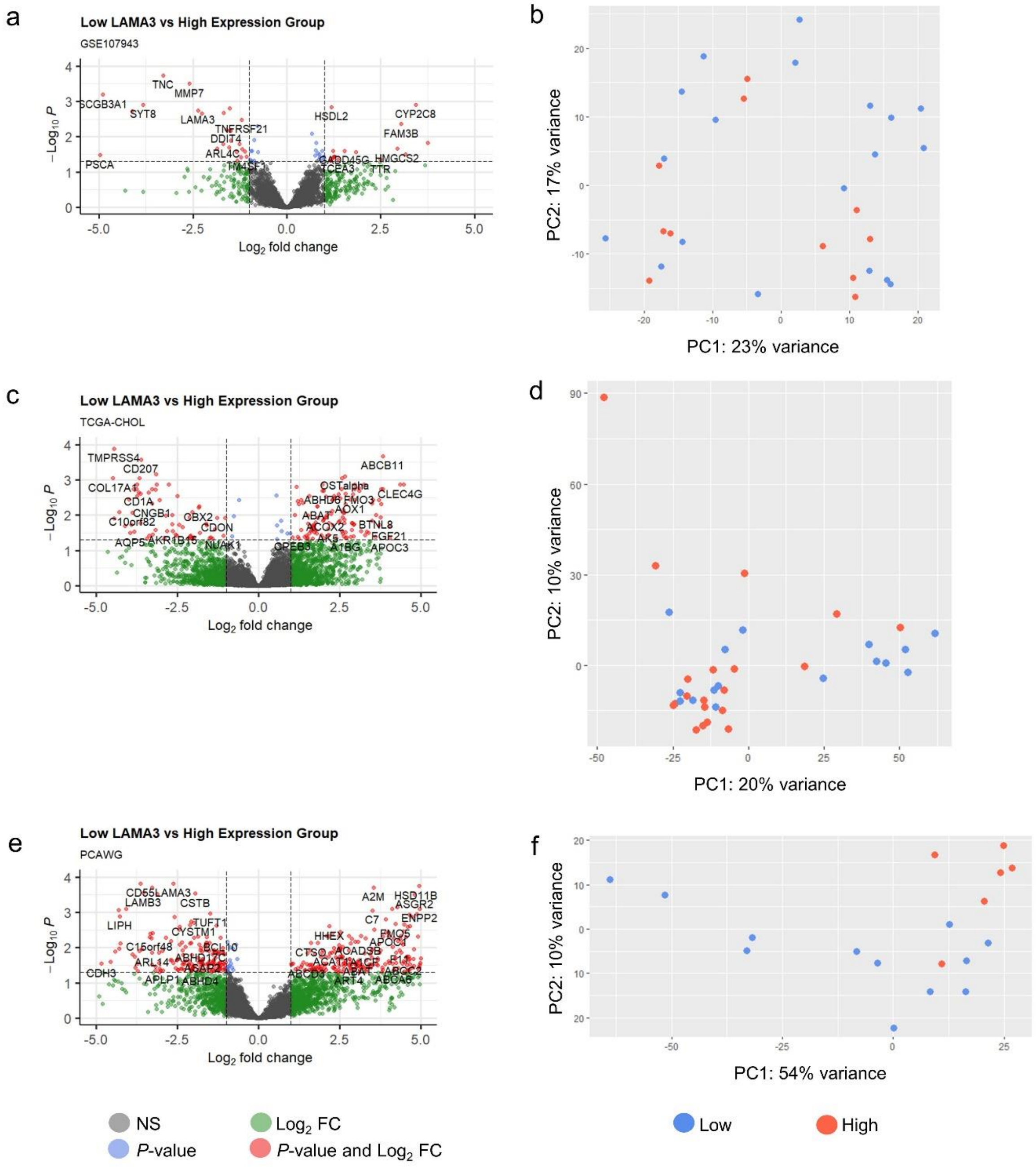

Supplementary Figure S7. Volcano plot of DEGs and PCA plot between low vs high LAMA3 group in the datasets. (**a, b**) GSE6107943; (**c, d**) TGCA-CHOL; (**e, f**) PCAWG. In the volcano plot, the x-axis indicates the fold-change (log-scaled); the y-axis indicates the *P*-values (log-scaled). The DEGs screened based on |Log2FC| of 2 with adjusted *P*-value < 0.05.

Supplementary Figure S8. Reduction of LAMA3 deposition in ECM of LAMA3 silenced HuCCA-1.

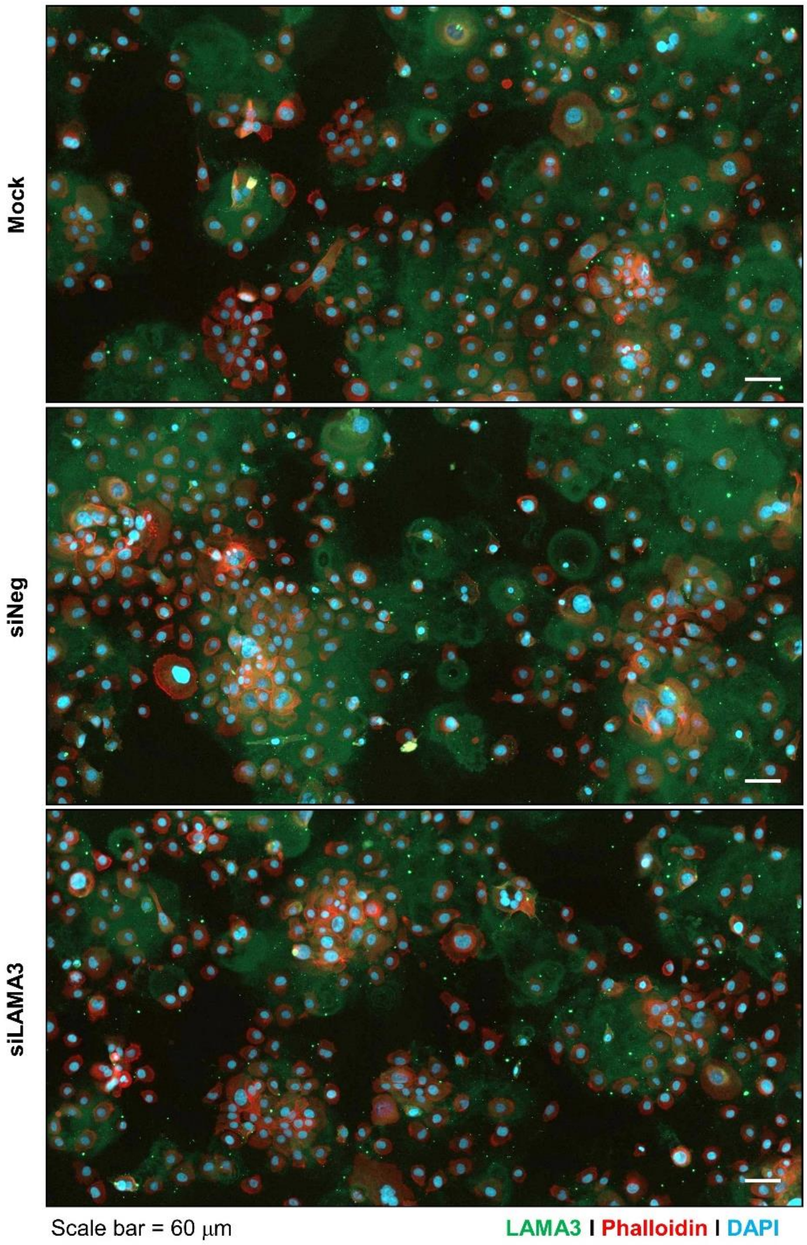

Supplementary Figure S8. Reduction of LAMA3 deposition in ECM of LAMA3 silenced HuCCA-1. Prior to LAMA3 knockdown for 24 hours, HuCCA-1 cells were pre-seeded and knocked down for 48 hours, following by immunofluorescence staining. Images were captured under 20 × magnification. Green stains for LAMA3 (MAB2144; R&D Systems) (RRID:AB_2133775). Red is phalloidin staining for F-actin and blue is DAPI staining for nucleus. Scale bar: 60 μm.

Supplementary Video S1. Related to Fig. 6b (left), representative video comparing cell migration in siNeg- and siLAMA3-treated HuCCA-1. 48-hour post siRNA transfection, migration speed of cells was collected by cell tracking in time-lapse every 15 min for 12 hours using an Operetta High-Content Imaging System (PerkinElmer) with a 40 × objective lens.

Supplementary Video S2. Related to Fig. 6b (right), representative video comparing cell migration in siNeg- and siLAMA3-treated KKU-213. 48-hour post siRNA transfection, migration speed of cells was collected by cell tracking in time-lapse every 15 min for 12 hours using an Operetta High-Content Imaging System (PerkinElmer) with a 40 × objective lens.
